# JA‐responsive R2R3‐type MYB transcription factor OsMYB4P confers broad‐spectrum antiviral immunity in rice

**DOI:** 10.1111/pbi.70246

**Published:** 2025-07-13

**Authors:** Mingmin Lu, Qianqian He, Guoda Wang, Qingqing Fan, Wenkai Yan, Kaibo Gu, Zihang Yang, Juan Du, Kaili Xie, Lulu Li, Hehong Zhang, Jianping Chen, Zongtao Sun, Yanjun Li

**Affiliations:** ^1^ State Key Laboratory for Quality and Safety of Agro‐Products, Key Laboratory of Biotechnology in Plant Protection of MARA, Key Laboratory of Green Plant Protection of Zhejiang Province, Institute of Plant Virology Ningbo University Ningbo China

**Keywords:** transcription factors, R2R3‐type MYB, jasmonic acid, plant viruses, antiviral defence

## Abstract

Jasmonic acid (JA) plays a critical role in antiviral defence in rice, where viral infection activates JA signalling by degrading Jasmonate ZIM domain (JAZ) proteins, thereby releasing transcription factors (TFs) to drive JA‐mediated defence gene expression. While the JA‐responsive TF OsMYC2 has been extensively studied in rice, the involvement of MYB TFs in JA signalling remains largely unexplored. In this study, we identified a novel JA‐responsive R2R3‐type MYB TF OsMYB4P. OsMYB4P functions as a transcriptional activator, activating the expression of the JA synthesis gene *CM‐LOX1*, and positively regulating rice sensitivity to JA. Additionally, OsMYB4P interacts with OsJAZs and is subject to transcriptional inhibition by OsJAZs. Overexpression of *OsMYB4P* (*OE‐OsMYB4P*) enhances rice resistance to viral infection, whereas knockout mutants (*osmyb4p*) exhibit increased susceptibility. Further studies revealed that viral proteins SP8 and P2, encoded by southern rice black‐streaked dwarf virus (SRBSDV) and rice stripe virus (RSV), respectively, disrupt OsMYB4P self‐interaction. This interference reduces OsMYB4P protein stability, transcriptional activation and DNA binding ability, thereby suppressing JA signalling and facilitating the viral infection. Overall, our findings demonstrate that the JA‐responsive R2R3‐type MYB TF OsMYB4P, as a direct target of JAZs, plays a key role in JA signalling and contributes to broad‐spectrum antiviral defence in rice.

## Introduction

Rice (*Oryza sativa* L.), the primary food source for more than half of the global population (Dobermann and Fairhurst, 2000), is frequently threatened by viral diseases during its growth, particularly insect‐borne diseases, such as southern rice black stripe dwarf disease (SRBSDD) and rice stripe disease (RSD), causing enormous losses in both yield and quality. SRBSDD is caused by the Southern rice black‐streaked dwarf virus (SRBSDV), a double‐stranded RNA (dsRNA) virus with a genome consisting of 10 dsRNA segments. SRBSDV belongs to the *Fijivirus* genus (family *Spinareoviridae*) and is transmitted in a persistent‐propagative manner by the white‐backed planthopper (WBPH, *Sogatella furcifera*). SRBSDV‐infected rice plants exhibit symptoms such as dark green leaves, severe dwarfing and increased tillering (Wei and Li, 2016; Zhou *et al*., 2013). RSD is caused by the rice stripe virus (RSV), a single‐stranded (ss) negative‐sense RNA virus that is a typical member of the *Tenuivirus* genus. The RSV genome comprises four ssRNA segments, designated as RNA1 to RNA4. RSV is transmitted through a circulative‐propagative manner by the small brown planthopper (SBPH, *Laodelphax striatellus*). RSV‐infected rice plants commonly exhibit chlorotic stripes or patches on their leaves (Wei *et al*., 2009; Xu *et al*., 2021).

To rapidly establish infection and adapt to their hosts, plant viruses utilize their small genomes to encode multifunctional viral proteins. These viral proteins then act as virulence factors, suppressing the host's immune system to facilitate viral infection, replication and movement. Our recent study revealed that two distinct rice viruses, SRBSDV and RSV, share a common pathogenic strategy, despite employing independently evolved viral protein effectors (Li *et al*., 2024). For instance, although SRBSDV SP8 and RSV P2 share no significant sequence similarity or conserved protein domains, both function as viral transcriptional repressors and target common conserved host factors. These targets are reported to mediate broad‐spectrum antiviral responses, including OsARF17 (Zhang *et al*., 2020), OsMYC2 (Li *et al*., 2021), SLR1 (Li *et al*., 2022), OsNPR1 (Zhang *et al*., 2023), OsNF‐YCs (Tan *et al*., 2024) and OsRAV15 (Zhang *et al*., 2025). Plant–virus interactions are complicated and multifaceted, involving a complex network of signalling pathways, gene expression changes and metabolic adjustments (Wang, 2015; Wang *et al*., 2020). Although many common targets of these two rice viruses have been reported, numerous potential host interaction factors involved in the shared targets of SRBSDV SP8 and RSV P2 remain to be explored. Further investigation is necessary to identify and understand these factors to gain deeper insights into the mechanisms of viral infection and host response.

In the complex signalling network of rice–virus interactions, the involvement of plant hormone pathways has been widely reported. These hormones include jasmonic acid (JA), salicylic acid (SA), auxin, brassinosteroid (BR) and abscisic acid (ABA) signalling pathways (He *et al*., 2017; Qin *et al*., 2020; Xie *et al*., 2018; Yang *et al*., 2020; Zhang *et al*., 2019a, 2019b). Among them, the broad‐spectrum antiviral function of JA is well‐studied and widely recognized. For example, exogenous application of JA enhances rice resistance to rice black‐streaked dwarf virus (RBSDV) (He *et al*., 2017; Zhang *et al*., 2019b). In addition, the resistance of certain rice varieties to SRBSDV is closely linked to the JA pathway, with activation of JA signalling being a key factor in the increased resistance of XI varieties to SRBSDV (Huang *et al*., 2024). Similarly, RSV infection activates the JA signalling in rice, enhancing rice's defence against further RSV invasion (Han *et al*., 2020). The RSV‐encoded coat protein (CP) acts as the major viral component that induces the JA signalling, resulting in JA accumulation and up‐regulation of the JA signalling marker gene *JAMYB*, which initiates the host defence network (Han *et al*., 2020; Yang *et al*., 2020). Furthermore, JA enhances the efficiency of RNA silencing pathways, strengthening RNAi‐based antiviral activity. Thus, JA signalling and RNA silencing work synergistically to promote rice antiviral defence (Yang *et al*., 2020). JA and its derivatives are a class of oxygenated lipid hormones widely found in plants. Their biosynthesis begins with α‐linolenic acid (α‐LeA, 18:3) in the chloroplast and involves multiple reactions catalysed by 13‐lipoxygenase (LOX), allene oxide synthase (AOS), allene oxide cyclase (AOC), 12‐oxophytodienoic acid (OPDA) reductase 3 (OPR3), acyl‐CoA oxidase 1 (ACX1) and JA‐resistant 1 (JAR1) to produce JA‐Ile (Delker *et al*., 2006; Fonseca *et al*., 2009; Liu *et al*., 2015; Wasternack and Song, 2017). Within the signalling cascades, transcriptional repressor Jasmonate ZIM domain (JAZ) protein plays a central role (Pauwels and Goossens, 2011). Upon sensing JA‐Ile, a ternary complex forms between JA‐Ile, receptor protein coronatine insensitive 1 (COI1) and JAZ repressor protein, leading to JAZ ubiquitination and degradation, thereby releasing and activating TFs (such as MYC2) to activate JA‐responsive gene expression (Dombrecht *et al*., 2007; Kazan and Manners, 2013; Wasternack and Song, 2017; Yan *et al*., 2018).

In addition to MYC2, other TFs such as MYB, NAC, ERF and WRKY are also involved in JA signalling. Among these, MYB transcription factors are one of the most widely distributed TF families in plants, playing essential roles in various biological processes, including plant development, secondary metabolism and responses to abiotic and biotic stresses (Cao *et al*., 2020; Dubos *et al*., 2010; Jiang and Rao, 2020). MYB TFs are characterized by a highly conserved N‐terminal MYB DNA binding domain (DBD), typically consisting of one to four imperfect repeats (R) of approximately 52 amino acid residues (Du *et al*., 2015). Based on the number and arrangement of the MYB domains, MYB TFs are classified into four subfamilies: 1R‐MYBs (R1/R2, R3‐MYB), 2R‐MYBs (R2R3‐MYB), 3R‐MYBs (R1R2R3‐MYB) and 4R‐MYBs (R1/R2‐MYB) (Dubos *et al*., 2010). While R1R2R3‐type MYBs are predominant in animals, R2R3‐type MYBs are the most prevalent in plants (Dubos *et al*., 2010; Martin and Paz‐Ares, 1997). It has been reported that R2R3‐type MYB TFs MYB21 and MYB24 are JA‐responsive TFs in *Arabidopsis*. JAZ proteins interact with MYB21 and MYB24 to suppress their transcriptional function. Upon JA signal perception, COI1 facilitates the ubiquitination and degradation of JAZ proteins, thereby releasing MYB21 and MYB24 to activate the expression of genes essential for JA‐regulated anther development and filament elongation (Huang *et al*., 2017; Song *et al*., 2011). Similarly, the R2R3‐type MYB TF MYB75 is also JA‐responsive, with JAZ proteins interacting to repress JA‐mediated anthocyanin accumulation and trichome initiation in *Arabidopsis* (Qi *et al*., 2011).

MYB TFs have also been reported to be involved in viral infections (Li *et al*., 2018; Slavokhotova *et al*., 2021; Sun *et al*., 2019; Yuan *et al*., 2021). However, reports on the involvement of JA‐responsive MYB TFs in regulating viral infections are rare. To date, only the R2R3‐type MYB TF *OsJAMYB* has been identified as a JA signalling marker gene involved in defence against RSV infection in rice (Yang *et al*., 2020). In this study, we identified a novel JA‐responsive MYB TF, OsMYB4P, in rice that participates in the JA pathway and confers rice broad‐spectrum antiviral immunity against SRBSDV and RSV. OsMYB4P functions as a transcriptional activator, targeting the JA biosynthetic gene *CM‐LOX1* and positively regulating JA signalling. Additionally, OsMYB4P interacts with OsJAZs, which suppress its transcriptional activity. Notably, we found that the viral proteins SRBSDV SP8 and RSV P2 interact with OsMYB4P, disrupting its self‐interaction and promoting its degradation. These viral proteins also collaborate with OsJAZ11 to synergistically suppress the transcriptional activity of OsMYB4P. This combined interference prevents the release and activation of OsMYB4P, thereby blocking the expression of JA‐responsive defence genes and promoting viral infection. In summary, this study identifies OsMYB4P as a novel JA‐responsive R2R3‐type MYB TF that acts as a direct target of OsJAZs to specifically mediate JA‐regulated broad‐spectrum antiviral immunity in rice.

## Results

### SP8 and P2 interact with R2R3‐type MYB transcription factor OsMYB4P

**Figure 1:**
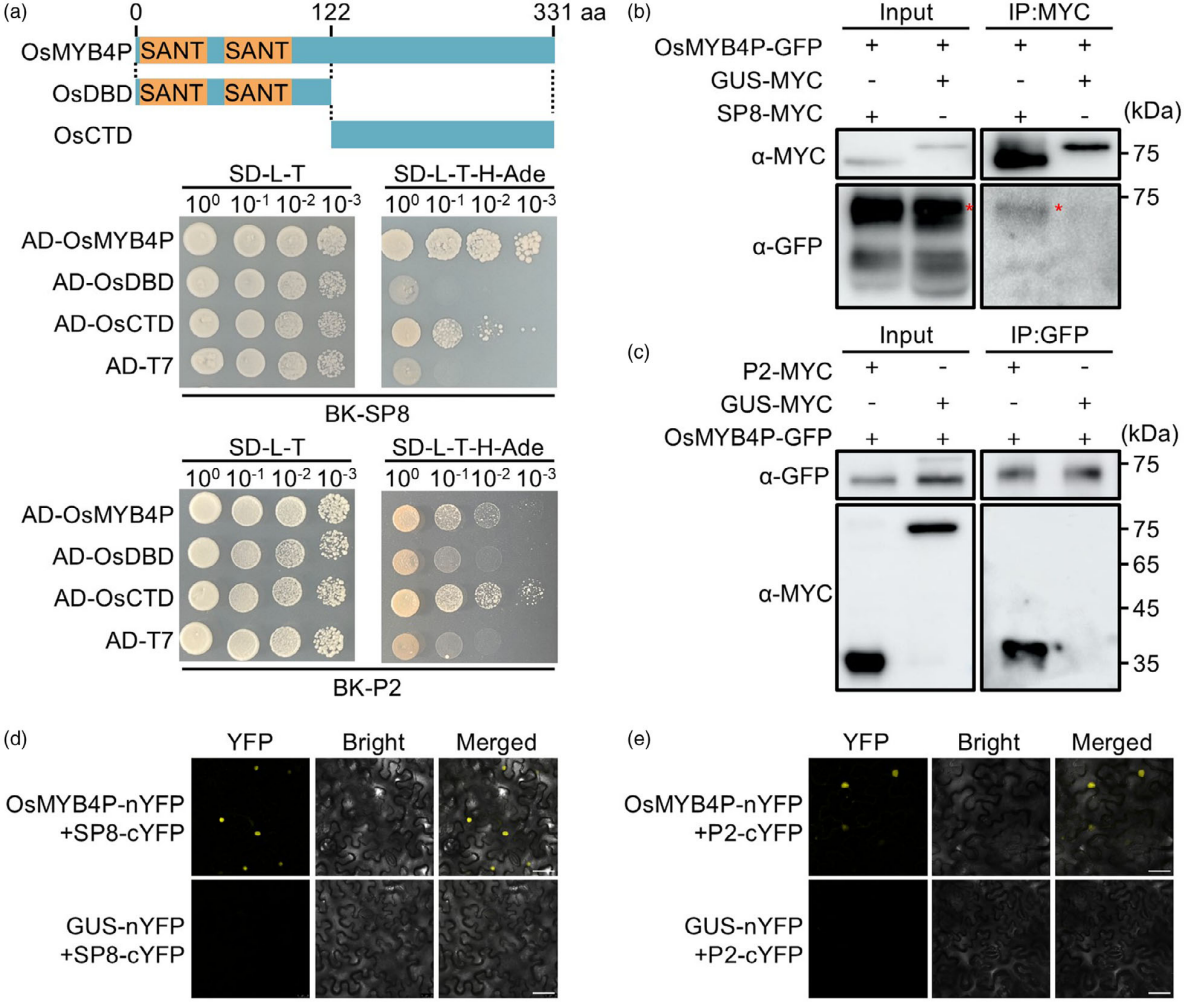
Viral proteins SP8 and P2 interact with OsMYB4P. (a) Schematic diagram of the OsMYB4P protein structure and its truncation variants (OsDBD and OsCTD) used in the Y2H assays. Y2H assays show that both SRBSDV SP8 and RSV P2 interact with the full‐length OsMYB4P and its C‐terminal domain (OsCTD). (b) Co‐IP assays show that SP8 interacts with OsMYB4P. OsMYB4P‐GFP was co‐expressed with SP8‐MYC or GUS‐MYC (negative control) in *N. benthamiana* leaves. The red asterisks point to the specific band. (c) Co‐IP assays show that P2 interacts with OsMYB4P. OsMYB4P‐GFP was co‐expressed with P2‐MYC or GUS‐MYC (negative control) in *N. benthamiana* leaves. (d) BiFC assays indicate the interaction between SP8 and OsMYB4P *in planta*. SP8‐cYFP was co‐expressed with OsMYB4P‐nYFP or GUS‐nYFP (negative control) in *N. benthamiana* leaves. Reconstituted YFP fluorescence signals were observed using confocal microscopy and photographed at 48 hpi. Scale bar, 50 μm. (e) BiFC assays indicate the interaction between P2 and OsMYB4P *in planta*. P2‐cYFP was co‐expressed with OsMYB4P‐nYFP or GUS‐nYFP (negative control) in *N. benthamiana* leaves. Reconstituted YFP fluorescence signals were observed using confocal microscopy and photographed at 48 hpi. Scale bar, 50 μm.

Our recent yeast two‐hybrid (Y2H) screening assay revealed that SRBSDV SP8 protein specifically interacted with the R2R3‐type MYB transcription factor OsMYB4P (Figure 1a). Notably, previous studies have shown that many host proteins are targeted by different viral proteins, reflecting a common pathogenic strategy among various viruses (Li *et al*., 2021, 2024). Consistent with this, our Y2H assay further confirmed that the RSV P2 protein also interacts with OsMYB4P (Figure 1a). Furthermore, conserved domain analysis revealed that the N‐terminal region of OsMYB4P contains two SANT domains with a conserved R2R3 repeats responsible for DNA binding. To determine which region of OsMYB4P is critical for interacting with viral proteins, we divided OsMYB4P into N‐terminal DNA binding domain (OsDBD) and the C‐terminal domain (OsCTD) (Figure 1a). Y2H assays showed that both SP8 and P2 specifically interact with the C‐terminal region, but not the N‐terminal region (Figure 1a). To further validate these interactions between viral proteins and OsMYB4P, we performed co‐immunoprecipitation (Co‐IP) and bimolecular fluorescence complementation (BiFC) assays *in planta*. The Co‐IP assays showed that SP8‐MYC and P2‐MYC, but not the control GUS‐MYC, specifically co‐precipitated with OsMYB4P‐GFP (Figure 1b,c). In the BiFC assay, OsMYB4P was fused to the N‐terminal part of YFP (OsMYB4P‐nYFP), while SP8 and P2 were fused to the C‐terminal part of YFP (SP8‐cYFP and P2‐cYFP). These constructs were co‐expressed in leaf cells of *Nicotiana benthamiana* (*N. benthamiana*). As shown in Figure 1d,e, co‐expression of OsMYB4P‐nYFP with either SP8‐cYFP or P2‐cYFP reconstituted the YFP signal in the nucleus, whereas no signal was observed in the negative controls, demonstrating that SP8 and P2 interact with OsMYB4P *in planta*. Together, these results indicate that SP8 and P2 specifically interact with the R2R3‐type MYB transcription factor OsMYB4P.

### OsMYB4P positively modulates rice antiviral defence

**Figure 2:**
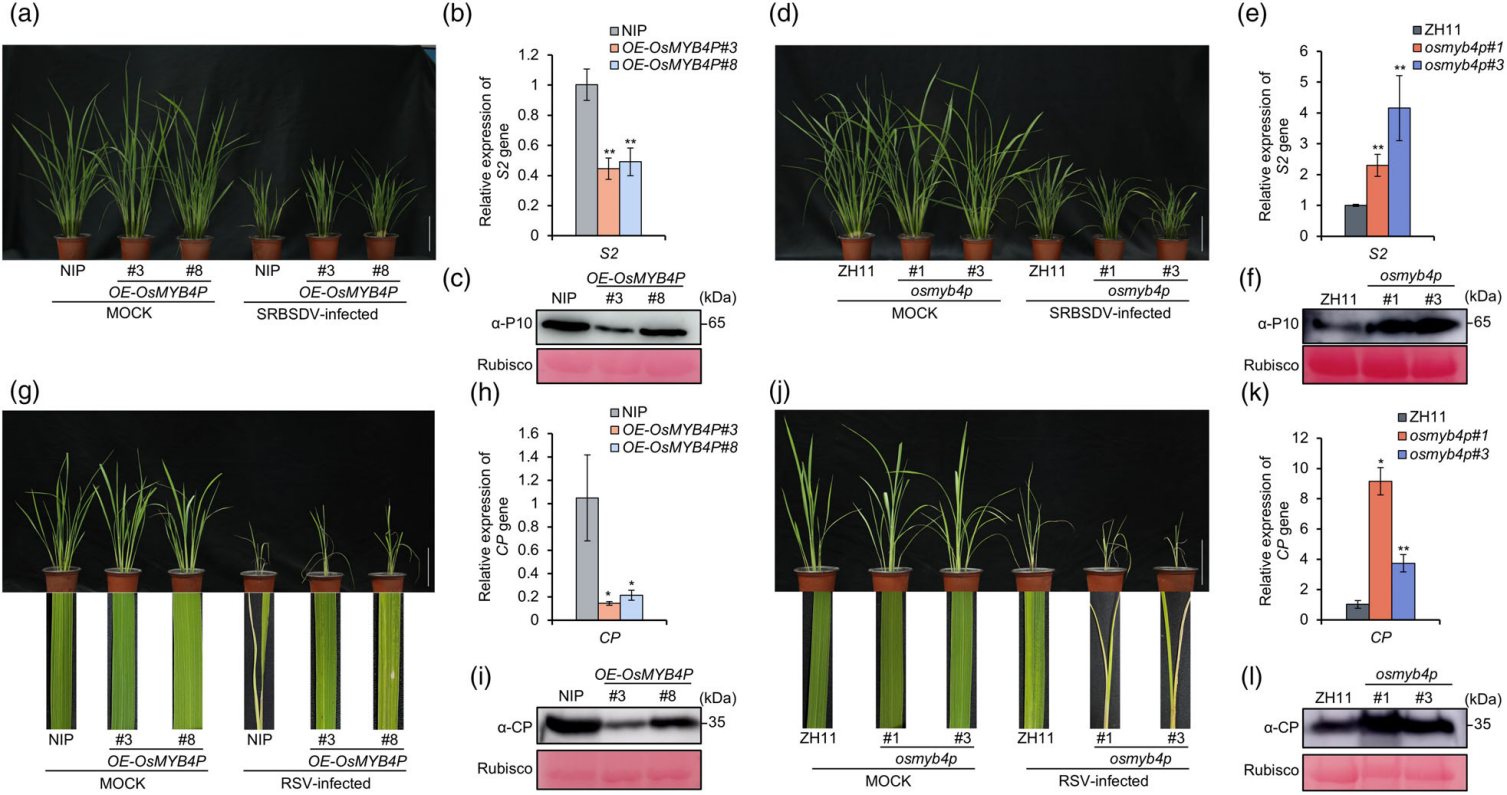
OsMYB4P positively modulates rice antiviral defences. (a, d) The symptoms of mock‐ and SRBSDV‐infected wild‐type (WT) (NIP) and *OE‐OsMYB4P* or wild‐type (WT) (ZH11) and *osmyb4p* plants. Scale bar, 10 cm. (b, e) Quantitative real‐time PCR (qRT‐PCR) analysis of SRBSDV RNA *S2* accumulation in infected plants. *OsUBQ5* was used as the internal reference gene. Data are shown as means ± SD of three biological replicates. Asterisks indicate significant differences. *P < 0.05, **P < 0.01, and ***P < 0.001 (Student's *t* test). (c, f) Western blot assays showing the accumulation levels of SRBSDV capsid protein P10 in infected plants. The Rubisco was used as a loading control. (g, j) The symptoms of mock‐ and RSV‐infected NIP and *OE‐OsMYB4P* or ZH11 and *osmyb4p* plants. Scale bar, 10 cm. (h, k) qRT‐PCR analysis of the relative expression of RSV *CP* in infected plants. *OsUBQ5* was used as the internal reference gene. Data are shown as means ± SD of three biological replicates. Asterisks indicate significant differences. *P < 0.05 and **P < 0.01 (Student's *t* test). (i, l) Western blot assays showing the accumulation levels of RSV CP protein in infected plants. The Rubisco was used as a loading control.

The interaction between OsMYB4P and viral proteins suggests that OsMYB4P may be involved in viral infection. To explore the effect of OsMYB4P on viral infection, we generated two CRISPR/Cas9 mutant lines, *osmyb4p*#1 and *osmyb4p*#3, in the *Zhonghua11* (ZH11) background (Figure S1a), as well as two overexpression (OE) lines, *OE‐OsMYB4P*#3 and *OE‐OsMYB4P*#8, in the *Nipponbare* (NIP) background (Figure S1b). Subsequently, these transgenic plants were inoculated with SRBSDV and RSV, and viral symptoms were monitored at 30 days and 20 days' post‐inoculation (dpi), respectively. Upon SRBSDV (a double‐stranded RNA virus) infection, *OsMYB4P OE* plants showed milder dwarfing symptoms compared to wild‐type NIP plants, whereas *osmyb4p* mutants showed more severe dwarfing symptoms than ZH11 plants (Figure 2a,d). Consistently, compared to their respective control plants, the accumulation of SRBSDV RNA *S2* and the capsid protein P10 was significantly reduced in *OsMYB4P OE* lines, while it was markedly increased in *osmyb4p* knockout lines (Figure 2b,c,e,f). A similar pattern was observed after challenging with RSV (a negative single‐stranded RNA virus): compared to NIP, *OsMYB4P OE* plants displayed milder yellow stripes symptoms on the leaves and reduced accumulation of viral RNA *CP* and the viral protein CP (Figure 2g,h,i). Conversely, *osmyb4p* mutants exhibited more severe disease symptoms and higher accumulation of viral RNA *CP* and the viral protein CP compared to ZH11 (Figure 2j,k,l). Together, these results indicate that OsMYB4P plays a positive role in enhancing rice antiviral defence against SRBSDV and RSV infection.

### Transcriptome analysis uncovers that JA pathway is involved in antiviral defence of OsMYB4P

**Figure 3:**
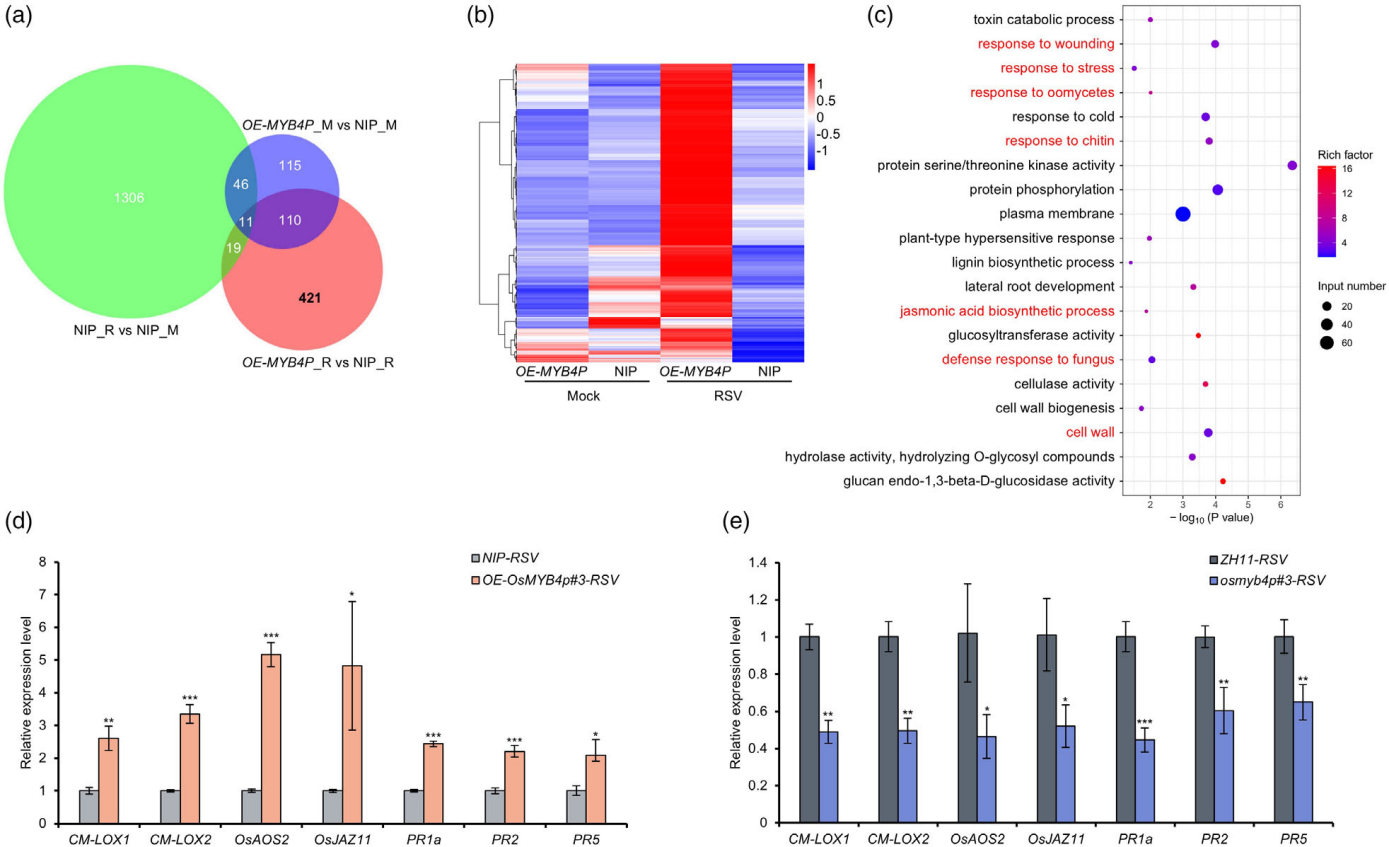
Transcriptomic analysis of gene expression profiles of *OE‐OsMYB4P* plants infected with RSV. (a) Venn diagram showing overlaps between up‐regulated differentially expressed genes (DEGs) obtained from *OE‐OsMYB4P*_Mock versus NIP_Mock, *OE‐OsMYB4P*_RSV versus NIP_RSV and NIP_RSV versus NIP_Mock. (b) Hierarchical clustering analysis of the 421 genes specifically up‐regulated in the *OE‐OsMYB4P*_RSV group. (c) Gene ontology (GO) analysis of the 421 genes, with JA‐ and defence response‐related GO terms highlighted in red. (d, e) qRT‐PCR analysis of *CM‐LOX1*, *CM‐LOX2*, *OsAOS2*, *OsJAZ11*, *PR1a*, *PR2* and *PR5* expression levels in *OE‐OsMYB4P* (d) and *osmyb4p* (e) mutant plants under RSV infection. Data are shown as means ± SD of three biological replicates. Asterisks indicate significant differences. *P < 0.05, **P < 0.01 and ***P < 0.001 (Student's *t* test).

To investigate how OsMYB4P positively modulates rice antiviral defence, we performed RNA‐seq analysis on *OE‐OsMYB4P*#3 and NIP plants infected with RSV. Principal component analysis (PCA) was performed to evaluate the reliability and suitability of the experimental system. The results indicated that different groups clustered separately, with minimal variation observed among biological replicates within the same group (Figure S2a). A threshold of *P*‐value <0.05 was applied to identify differentially expressed genes (DEGs). Bioinformatics analysis revealed 1382, 561, and 282 differentially up‐regulated genes in the comparisons of NIP_RSV versus NIP_Mock, *OE‐OsMYB4P*_RSV versus NIP_RSV and *OE‐OsMYB4P*_Mock versus NIP_Mock, respectively (Figure 3a). Venn diagram analysis revealed that 75% of these genes (421/561) were specifically up‐regulated in RSV‐infected *OE‐OsMYB4P* plants (Figure 3a). Hierarchical clustering analysis further confirmed that these 421 genes were uniquely activated in *OE‐OsMYB4P* plants compared to NIP plants after RSV infection (Figure 3b). Gene Ontology (GO) analysis of 421 up‐regulated genes revealed significant enrichment in categories related to defence response, response to stress, response to wounding, jasmonic acid biosynthetic process, cell wall and other disease resistance‐related pathways (Figure 3c). Among these, the JA biosynthetic process included genes such as *CM‐LOX1*, *OsOPR4*, *OsOPR5* and *LOC_Os03g53010* (Figure S2b). However, our qRT‐PCR results confirmed the induction of several other JA pathway genes in RSV‐infected *OE‐OsMYB4P*#3, including *CM‐LOX2*, *OsAOS2* and *OsJAZ11* (Figure 3d). Although the transcript levels of *CM‐LOX2*, *OsAOS2* and *OsJAZ11* did not show significant changes in the RNA‐Seq data (Table S2), qRT‐PCR analysis showed increased levels of these genes in RSV‐infected *OE‐OsMYB4P*#3. This inconsistency may be due to the low sequencing depth of RNA sequencing. Additionally, disease resistance‐related genes downstream of the JA pathway, including *PR1a* (pathogenesis‐related protein‐1a), *PR2* (β‐1,3‐glucanase) and *PR5* (thaumatin‐like protein), were also found to be up‐regulated in RSV‐infected *OE‐OsMYB4P*#3 (Figure 3d). In contrast to *OE‐OsMYB4P*, these genes were down‐regulated in RSV‐infected *osmyb4p* (Figure 3e). Taken together, these results suggest that OsMYB4P regulates the JA pathway for antiviral defence.

### OsMYB4P, as a transcriptional activator, positively regulates the JA pathway

**Figure 4:**
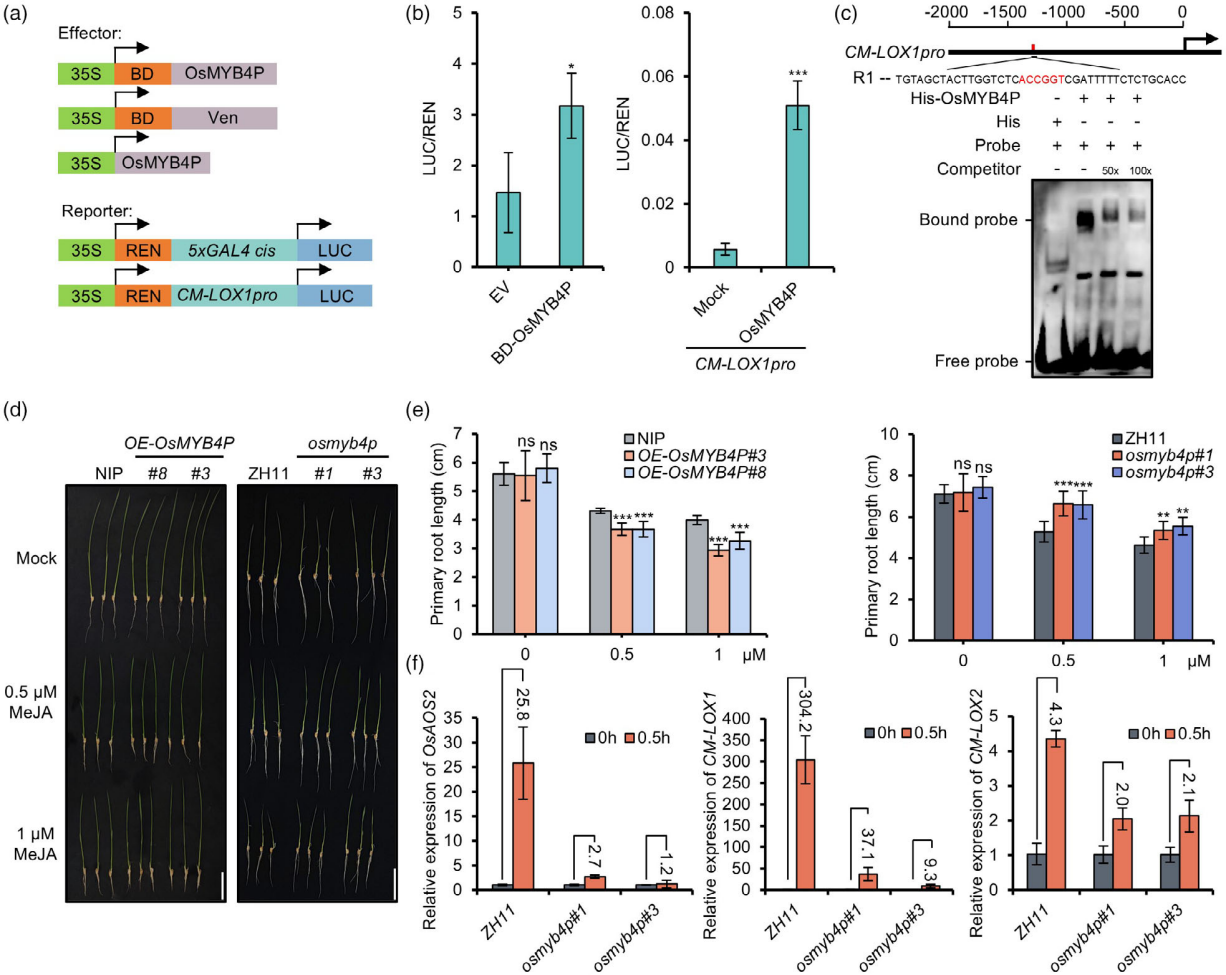
OsMYB4P functions as a transcriptional activator to regulate the JA pathway. (a) Schematic diagram of the effectors and reporters used in the GD system and dual‐luciferase transient transcriptional activity assay. The reporter gene *LUC* was driven by the *CM‐LOX1* and *5 × GAL4* promoters, while the effectors BD‐VEN (negative control), BD‐OsMYB4P and OsMYB4P were driven by the CaMV 35S promoter. Firefly luciferase (LUC) and renilla luciferase (REN) activities were measured, and the LUC/REN ratio was calculated to assess transcriptional activity. (b) OsMYB4P functions as a transcriptional activator. The *CM‐LOX1* promoter was activated by OsMYB4P *in planta*. Data are shown as means ± SD of three biological replicates. Asterisks indicate significant differences. *P < 0.05 and ***P < 0.001 (Student's *t* test). (c) EMSA assay showing that OsMYB4P binds to the ACCGGT motif of the *CM‐LOX1* promoter. The purified His‐OsMYB4P protein bound biotin‐labelled DNA probes, with competition observed using 50X and 100X excess unlabeled probes. (d) Phenotypes of *OE‐OsMYB4P* and *osmyb4p* mutant plants grown in rice nutrient solution containing 0, 0.5 and 1 μM MeJA for 5 days. Scale bar, 4 cm. (e) Quantification of primary root length in the indicated plants following MeJA treatment. Data are derived from at least 5 seedlings per sample. Values are mean ± SD. Asterisks indicate significant differences. **P < 0.01 and ***P < 0.001 (Student's *t* test). (f) qRT‐PCR analysis of JA pathway genes (*OsAOS2*, *CM‐LOX1* and *CM‐LOX2*) in 10‐day‐old *osmyb4p* mutant plants compared with 10‐day‐old ZH11 under 50 μM MeJA treatment. Representative data from three biological replicates are shown. Values are mean ± SD.

Previous studies have found that OsMYB4P is localized in the nucleus and acts as a transcriptional activator, regulating phosphate acquisition in rice (Yang *et al*., 2014). Our results are consistent with these findings. Fluorescence analysis of GFP‐tagged OsMYB4P revealed that OsMYB4P‐GFP was localized in the nucleus (Figure S3a). Furthermore, using the GAL4 DNA‐binding domain (GD) system, we verified that OsMYB4P functions as a transcriptional activator, as it significantly increased LUC activity compared with the vector alone (Figure 4a,b). The homologue of OsMYB4P in *Arabidopsis thaliana*, AtMYB60, has been reported to regulate the expression of JA biosynthesis‐related *13‐LOX* genes (Abdullah‐Zawawi *et al*., 2021; Simeoni *et al*., 2022). This insight prompted us to explore whether OsMYB4P also regulates the transcription of *13‐LOX* genes in rice. For validation, we chose the *13‐LOX* gene, *CM‐LOX1*, which is not only significantly induced by RSV infection (Yang *et al*., 2020), but also shows marked up‐regulation in RSV‐infected *OE‐OsMYB4P*#3 as indicated by RNA‐Seq (Figure S2b). Using a dual‐luciferase transient transcriptional activity assay, we confirmed that OsMYB4P expression activates the transcription of *CM‐LOX1*, but not the negative control (Figure 4a,b). To further confirm the direct binding of OsMYB4P to the *CM‐LOX1* promoter, we performed an electrophoretic mobility shift assay (EMSA) using His‐tagged OsMYB4P protein. The results showed that OsMYB4P specifically bound to the probe containing the *CM‐LOX1* promoter *cis*‐element (ACCGGT) (Figure S3b), and this binding was effectively competed by an unlabeled probe (Figure 4c). Additionally, qRT‐PCR assays confirmed that *CM‐LOX1* expression was significantly up‐regulated in *OE‐OsMYB4P*#3 plants, accompanied by an increase in JA content (Figure S4a,b). Taken together, these findings indicate that OsMYB4P directly binds to the *CM‐LOX1* promoter to activate its expression.

To further clarify the role of OsMYB4P in the JA pathway, we tested the sensitivity of *OsMYB4P*‐transgenic plants to methyl jasmonate (MeJA) under hydroponic conditions. JA treatment typically inhibits root growth, with a stronger inhibitory effect in plants with activated JA signalling. After 5 days of treatment with 0.5 μM or 1 μM MeJA, the root lengths of *OsMYB4P*‐transgenic plants and their corresponding wild‐type controls were measured. The results showed that root growth was significantly more inhibited in *OE‐OsMYB4P* plants than in NIP, while *osmyb4p* mutants showed less inhibition compared to ZH11, indicating that overexpression of *OsMYB4P* activated JA signalling in rice (Figure 4d,e). Additionally, qRT‐PCR analysis showed that, compared to wild‐type seedlings, MeJA treatment significantly induced the expression of several JA pathway‐related genes in ZH11, including *OsAOS2*, *CM‐LOX1* and *CM‐LOX2*. However, in *osmyb4p* mutants, this induction effect was significantly diminished (Figure 4f). Moreover, MeJA treatment rapidly induced *OsMYB4P* expression within a short time, indicating that OsMYB4P is a JA‐responsive MYB transcription factor (Figure S4c). Together, these findings indicate that *OsMYB4P* functions as a JA‐responsive transcriptional activator and serves as a positive regulator of the JA pathway in rice.

### OsMYB4P interacts with rice JAZ repressor proteins

**Figure 5:**
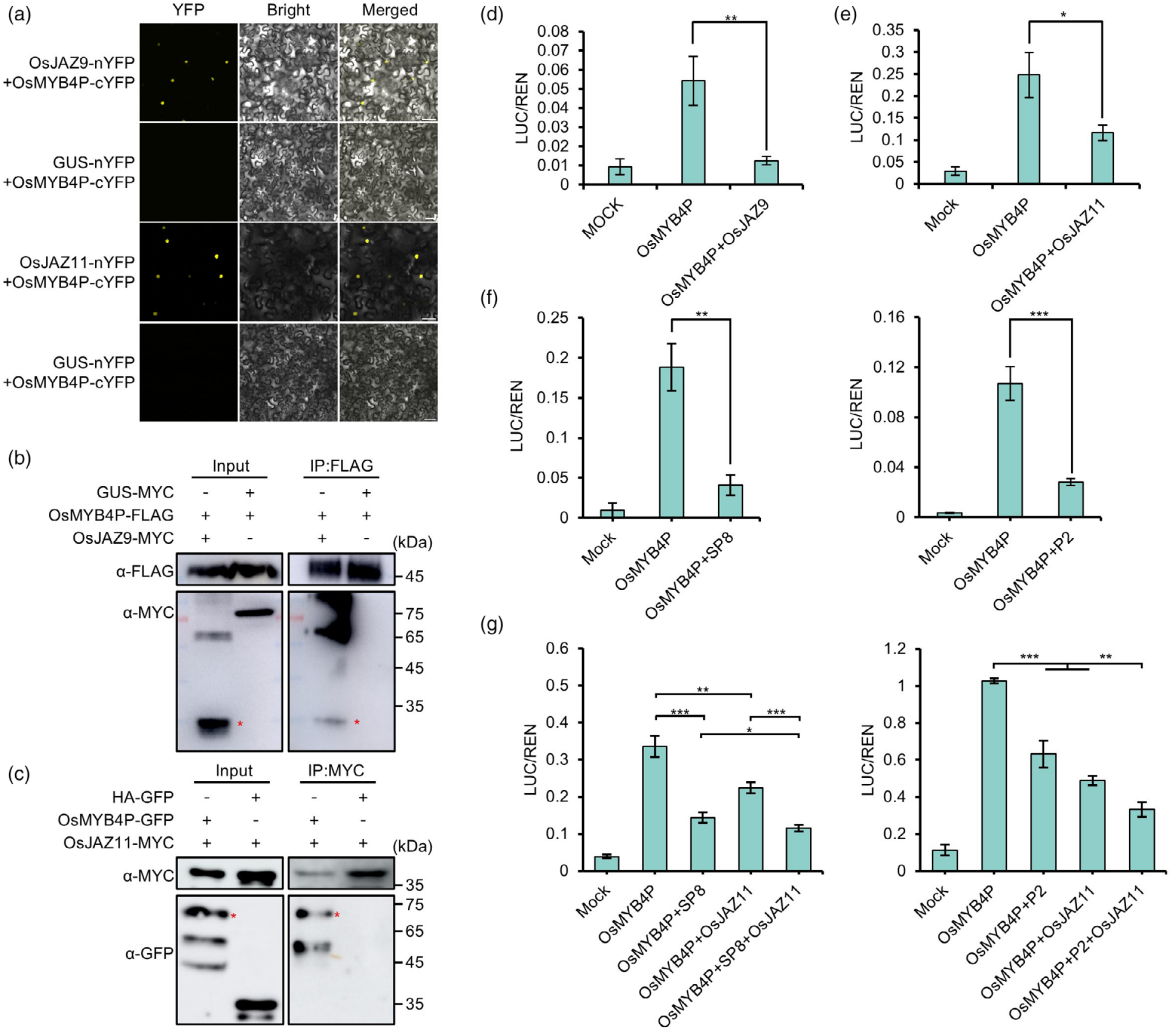
OsJAZs interact with OsMYB4P and inhibit the transcriptional activation activity of OsMYB4P. (a) Analysis of the interactions between OsJAZs and OsMYB4P by BiFC assays. BiFC assays indicate that OsMYB4P interacts specifically with OsJAZ9 and OsJAZ11 *in planta*. Scale bar, 50 μm. (b, c) Co‐IP assays show that OsMYB4P interacts with OsJAZ9 (b) and OsJAZ11 (c). OsMYB4P‐FLAG was co‐expressed with the OsJAZ9‐MYC or GUS‐MYC (negative control) in *N. benthamiana* leaves. OsJAZ11‐MYC was co‐expressed with the OsMYB4P‐GFP or HA‐GFP (negative control) in *N. benthamiana* leaves. The red asterisks point to the specific band. (d–f) OsJAZ9 (d), OsJAZ11 (e), SP8 and P2 (f) repress the transcription activity of *OsMYB4P* on the *CM‐LOX1* promoter. The *CM‐LOX1pro::LUC* reporter was co‐expressed with the indicated effectors in *N. benthamiana* leaves and measured at 48 hpi. Relative luciferase activities were analysed by the ratio LUC/REN. Data are shown as means ± SD of three biological replicates. Asterisks indicate significant differences. *P < 0.05, **P < 0.01 and ***P < 0.001 (Student's *t* test). (g) SP8 and P2 interact with OsJAZ11 to synergistically suppress the transcriptional activation of *OsMYB4P*. The *CM‐LOX1pro::LUC* reporter was co‐expressed with the indicated effectors in *N. benthamiana* leaves and measured at 48 hpi. Relative luciferase activities were analysed by the ratio LUC/REN. Data are shown as means ± SD of three biological replicates. Asterisks indicate significant differences. *P < 0.05, **P < 0.01 and ***P < 0.001 (Student's *t* test).

A common feature of JA‐responsive TFs is their interaction with JAZ proteins, which suppress the transcriptional activity of TFs to maintain JA homeostasis. For example, the transcriptional activity of JA‐responsive OsMYC2 and JAMYB in rice, as well as MYB21, MYB24 and MYB75 in *Arabidopsis*, relies on interactions with endogenous JAZ repressor proteins. Based on this, we hypothesized that JAZ proteins might also suppress the transcriptional activity of JA‐responsive OsMYB4P. It has been reported that JAZ proteins inhibit the activity of TFs through direct protein–protein interactions (Pauwels and Goossens, 2011). To explore the potential interaction between OsMYB4P and rice JAZ proteins, a Y2H screening assay was performed, revealing no detectable interaction between OsMYB4P and rice JAZ proteins (Figure S5). However, the results of BiFC experiments confirmed that OsMYB4P interacts with OsJAZ4, OsJAZ6, OsJAZ9 and OsJAZ11 *in planta* (Figure 5a; Figure S6a). Furthermore, Co‐IP assays were performed for further validation, confirming that OsMYB4P interacts specifically with OsJAZ9 and OsJAZ11(Figure 5b,c; Figure S6b,c). These results indicate that OsMYB4P functions as a direct target of OsJAZs. To determine whether the interaction between OsJAZs and OsMYB4P leads to inhibition of the transcriptional activation activity of OsMYB4P, we performed dual‐luciferase transient transcriptional activity assays. We co‐transformed *35S:OsMYB4P* and *CM‐LOX1pro::LUC* reporter along with effector *35S:OsJAZ9* and *35S:OsJAZ11* into *N. benthamiana* leaves and measured the results at 48 h post‐infiltration (hpi) (Figure S6d). The activation of the *CM‐LOX1pro::LUC* reporter was significantly repressed by *35S:OsJAZ9* and *35S:OsJAZ11* (Figure 5d,e). These results suggest that OsJAZs interact with OsMYB4P to attenuate its transcriptional activity.

**Figure 6:**
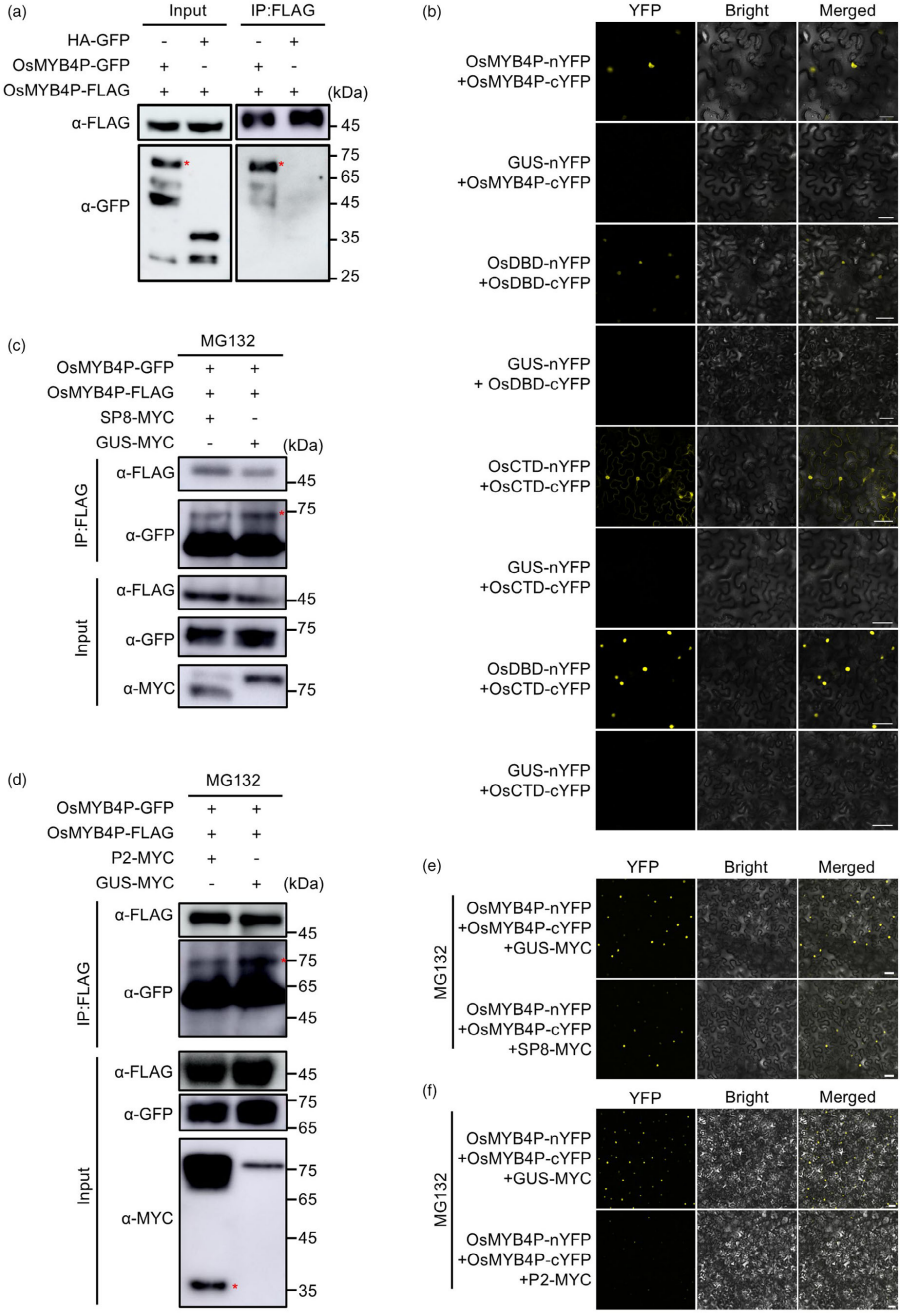
Viral proteins SP8 and P2 interfere with OsMYB4P self‐interaction. (a) Co‐IP assays show the self‐interaction of OsMYB4P. OsMYB4P‐FLAG was co‐expressed with the OsMYB4P‐GFP or HA‐GFP (negative control) in *N. benthamiana* leaves. The red asterisks point to the specific band. (b) BiFC assays show that the OsMYB4P, OsDBD and OsCTD can self‐interact. Additionally, OsDBD and OsCTD also interact *in planta*. GUS‐nYFP served as a negative control. Reconstituted YFP fluorescence signals were observed using confocal microscopy and photographed at 48 hpi. Scale bar, 50 μm. (c, d) The effects of SP8 and P2 on the OsMYB4P self‐interaction were evaluated through the competitive Co‐IP assays. OsMYB4P‐FLAG and OsMYB4P‐GFP were co‐infiltrated into *N. benthamiana* leaves along with SP8‐MYC (c) or P2‐MYC (d). GUS‐MYC served as negative control. Total proteins were extracted and immunoprecipitated using anti‐FLAG magnetic beads. The red asterisks point to the specific band. (e and f) Reconstituted YFP fluorescence signals from OsMYB4P‐cYFP and OsMYB4P‐nYFP were observed in *N. benthamiana* cells co‐expressing SP8‐MYC (e) or P2‐MYC (f). GUS‐MYC served as a negative control. The co‐infiltrated leaves were treated with MG132 (50 μM) at 24 hpi and then the images were photographed using confocal microscopy 24 h later. Scale bar, 50 μm.

### SP8 and P2 synergize with OsJAZ11 to attenuate transcriptional activity of OsMYB4P

Our previous studies identified SP8 and P2 as independently evolved but functionally conserved viral transcription suppressors in plant RNA viruses (Li *et al*., 2021). Both SP8 and P2 interact with the transcriptional activator OsMYB4P, suggesting that in the arms race between rice and viruses, these viral proteins may modulate the transcriptional activation of OsMYB4P to facilitate viral infection. To test this hypothesis, we performed dual‐luciferase transient transcriptional activity assays. Co‐expression of *35S:SP8* or *35S:P2* effectors with 3*5S:OsMYB4P* and *CM‐LOX1pro::LUC* reporter in *N. benthamiana* showed that SP8 and P2 markedly suppressed OsMYB4P‐mediated activation of the reporter (Figure 5f; Figure S6d). These findings indicate that SP8 and P2 directly repress the transcriptional activation of OsMYB4P.

Furthermore, our earlier research confirmed that SP8 and P2 interact with OsJAZ proteins, including OsJAZ11, and cooperatively suppress the OsMYC3‐mediated transcriptional programme, thereby synergistically attenuating JA signalling (Li *et al*., 2021). To investigate whether SP8 and P2 interact with OsJAZ11 to synergistically suppress the transcriptional activity of OsMYB4P, we conducted another dual‐luciferase transient transcriptional activity assay. Co‐expression of *35S:SP8*, *35S:P2* and *35S:OsJAZ11* amplified their suppression of OsMYB4P‐mediated activation of *CM‐LOX1pro::LUC* (Figure 5g; Figure S6d). These findings support the hypothesis that SP8 and P2 associate with OsJAZ11 to synergistically inhibit OsMYB4P's transcriptional activity.

### SP8 and P2 disrupt the self‐interaction of OsMYB4P

In many eukaryotic TF gene families, TFs often require physical interactions with themselves or other molecules within the same family to form functional dimers or oligomers, thereby activating transcription (Amoutzias *et al*., 2008; Li *et al*., 2014; Lian *et al*., 2017; Zhang *et al*., 2020; Zhao *et al*., 2024; Zheng *et al*., 2023). We confirmed that OsMYB4P underwent self‐interaction through Co‐IP and BiFC assays (Figure 6a,b). However, due to its self‐activation property, the self‐interaction of OsMYB4P could not be validated using the Y2H system (Figure S7a). Further analysis using truncated forms revealed that both the N‐terminal and C‐terminal domains of OsMYB4P could mediate self‐interaction, and interaction also occurred between the N‐terminal and C‐terminal domains (Figure 6b; Figure S7b). To further characterize the oligomeric state of OsMYB4P upon self‐interaction, we performed native PAGE analysis. The results revealed that OsMYB4P forms higher‐order oligomers (Figure S8). These findings indicate that OsMYB4P can interact with itself and assemble into an oligomeric complex.

To further investigate whether SP8 and P2 affected the self‐interaction of OsMYB4P, we validated this using competitive Co‐IP and BiFC assays. First, the competitive Co‐IP assays showed that OsMYB4P‐GFP co‐immunoprecipitated with OsMYB4P‐FLAG in the presence of the negative control GUS‐MYC (Figure 6c,d). However, the addition of SP8‐MYC or P2‐MYC significantly reduced the binding affinity between OsMYB4P proteins (Figure 6c,d). Consistent with this observation, we further conducted BiFC assays to assess the impact of viral proteins on OsMYB4P self‐interaction. BiFC analysis revealed that co‐expression of OsMYB4P‐cYFP and OsMYB4P‐nYFP in tobacco leaves produced a reconstituted a YFP fluorescence signal (Figure 6e,f). However, in the presence of SP8‐MYC or P2‐MYC, the fluorescence signal was markedly reduced (Figure 6e,f). Collectively, these results demonstrate that both SP8 and P2 disrupt the interaction between OsMYB4P proteins, interfering with its oligomerization.

### SP8 and P2 affect the stability and DNA binding ability of OsMYB4P by disrupting its self‐interaction

**Figure 7:**
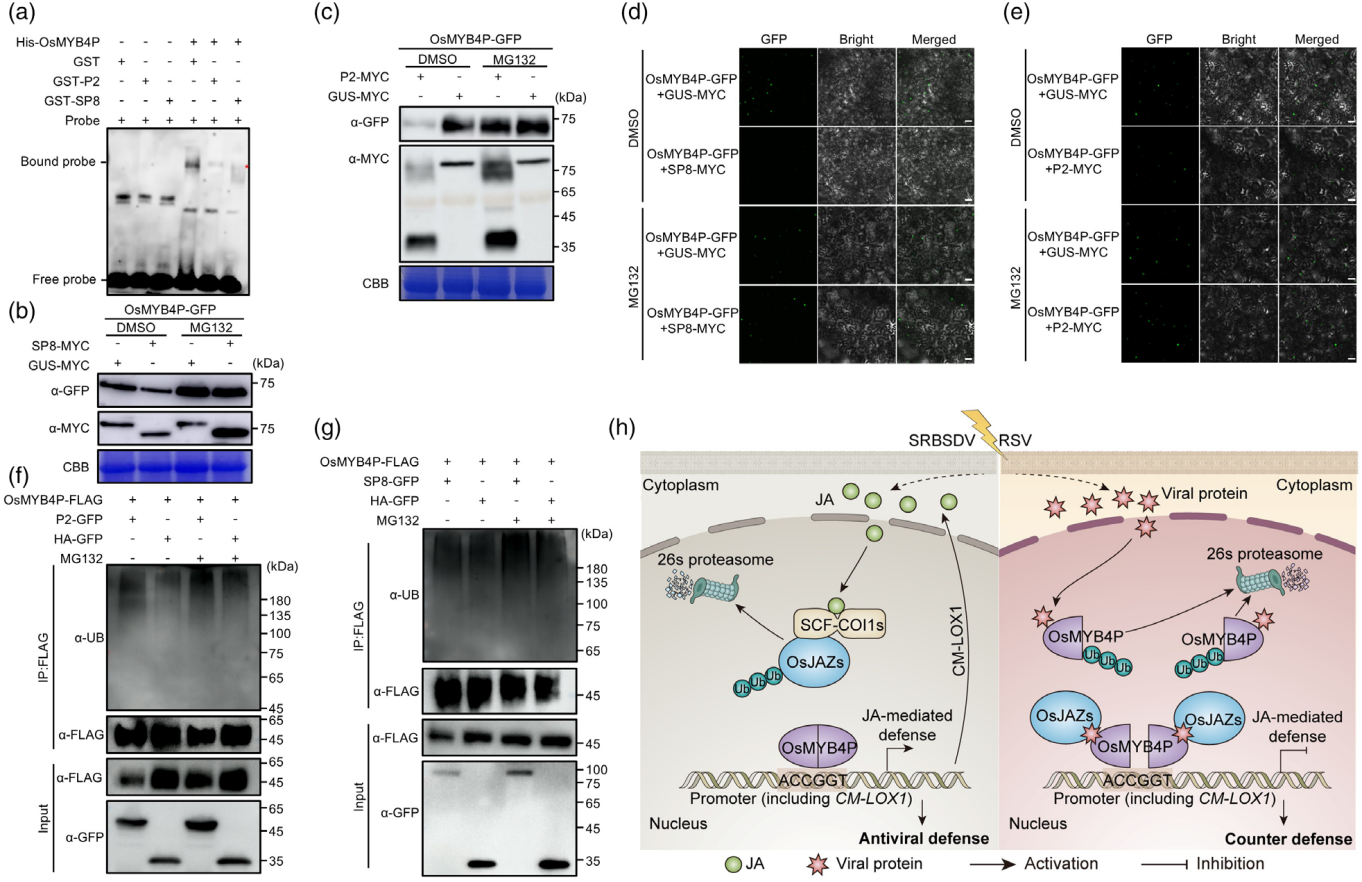
Viral proteins SP8 and P2 interfere with the DNA binding ability and protein stability of OsMYB4P. (a) The effects of SP8 and P2 on the DNA binding ability of OsMYB4P were evaluated using EMSA. Recombinant His‐OsMYB4P was incubated with GST‐SP8, GST‐P2 or GST (negative control) along with the *CM‐LOX1* probe. The samples were then separated on a native agarose gel, with red asterisks indicating the specific band. (b, c) SP8 and P2 induce OsMYB4P degradation, which is blocked by the proteasome inhibitor MG132. OsMYB4P‐GFP was co‐expressed with SP8‐MYC (b) or P2‐MYC (c) in *N. benthamiana* leaves by agroinfiltration. Leaves were treated with MG132 (50 μM) or DMSO at 24 hpi and then harvested for Western blotting 24 h later. GUS‐MYC served as the negative control. CBB staining was used as a loading control. (d, e) BiFC assays confirmed that SP8 and P2 affect the accumulation of OsMYB4P *in planta*. OsMYB4P‐GFP was co‐expressed with SP8‐MYC (d) or P2‐MYC (e) in *N. benthamiana* leaves. Co‐infiltrated leaves were treated with MG132 (50 μM) or DMSO at 24 hpi and then photographed using confocal microscopy at 48 hpi. (f, g) Ubiquitination assays confirm that OsMYB4P is ubiquitinated, and its ubiquitination level is enhanced in the presence of P2 (f) or SP8 (g). These assays were conducted in *N. benthamiana* leaves co‐expressing OsMYB4P‐FLAG with either HA‐GFP, P2‐GFP (f) or SP8‐GFP (g). OsMYB4P was enriched using FLAG magnetic beads, followed by Western blot analysis with anti‐Ub antibodies. (h) A proposed working model illustrating how the viral proteins suppress OsMYB4P‐mediated antiviral immunity in rice. Left panel: viral infection induces an increase in JA levels in rice plants. JA binds to and promotes the degradation of OsJAZs, thereby releasing OsMYB4P, which activates JA signalling to enhance the host's antiviral defence. Right panel: to counteract the host's antiviral defence, viral proteins disrupt OsMYB4P self‐interaction. This disruption reduces the protein stability, transcriptional activation, and DNA‐binding ability of OsMYB4P, thereby suppressing JA signalling and facilitating viral infection.

The oligomeric state of TFs is critical for their transcriptional activity or DNA binding ability (Lian *et al*., 2017; Liu *et al*., 1999). To further investigate whether viral proteins interfering with OsMYB4P self‐interaction affect its transcriptional DNA binding ability, we conducted EMSA. Previous studies confirmed that OsMYB4P directly binds to the *CM‐LOX1* promoter containing the ACCGGT motif. Based on this, we added SP8 or P2 to the EMSA reaction system to evaluate their impact. The results showed that the presence of SP8 and P2 significantly impaired the DNA binding ability of OsMYB4P (Figure 7a). These results suggest that viral proteins disrupt the self‐interaction of OsMYB4P, impairing its transcriptional DNA binding ability.

Typically, oligomers promote protein stability, while monomers are more prone to degradation. We then wondered whether the stability of OsMYB4P was directly affected by SP8 and P2 disrupting its self‐interaction in *planta*. To test this, we expressed OsMYB4P‐GFP with or without co‐expression of the viral proteins in tobacco leaves and found that the presence of SP8 or P2 led to a significant reduction in OsMYB4P protein levels. However, this reduction was reversed upon the addition of the 26S proteasome inhibitor MG132 (Figure 7b,c). Moreover, despite the changes in protein levels, the transcript levels of OsMYB4P remained unchanged (Figure S9). Similarly, confocal assays revealed that in the presence of SP8 and P2, the GFP‐tagged OsMYB4P exhibited weaker fluorescence signals, which were restored upon the addition of MG132 (Figure 7d,e). These results suggest that the viral proteins may destabilize OsMYB4P by interfering with its self‐interaction.

The degradation via the 26S proteasome pathway suggests that OsMYB4P undergoes ubiquitination. We next investigated whether OsMYB4P is ubiquitinated and whether viral proteins affect its ubiquitination level. Ubiquitination assays were performed in *N. benthamiana* leaves co‐expressing OsMYB4P‐FLAG with either HA‐GFP, P2‐GFP or SP8‐GFP (Figure 7f,g). Total proteins were extracted, and OsMYB4P was enriched using FLAG magnetic beads with or without MG132 treatment. Subsequent Western blot analysis with anti‐Ub antibodies confirmed the ubiquitination of OsMYB4P. Notably, in the presence of P2 or SP8, the ubiquitination of normalized OsMYB4P was markedly enhanced (Figure 7f,g). These results suggest that viral proteins promote the ubiquitination and degradation of OsMYB4P.

## Discussion

Most plant species encode over 100 R2R3‐type MYB proteins. For example, *Arabidopsis* and rice contain 126 and 117 R2R3‐type MYB genes, respectively (Stracke *et al*., 2001; Zhang *et al*., 2024). R2R3‐type MYB proteins have been found to participate in diverse processes, especially in plant stress responses (Dubos *et al*., 2010; Kang *et al*., 2022; Millard *et al*., 2019; Roy, 2016). In this study, we identified an R2R3‐type MYB TF, OsMYB4P, which was previously reported to regulate phosphate acquisition. Our findings further reveal its role in broad‐spectrum antiviral immunity against SRBSDV and RSV in rice. Additionally, we confirm earlier reports that OsMYB4P localizes in the nucleus and functions as a transcriptional activator. Beyond its role in phosphate acquisition, OsMYB4P transcriptionally activates the expression of the JA synthesis gene *CM‐LOX1*, positively regulating rice sensitivity to JA. Moreover, OsMYB4P interacts with JAZ transcriptional repressors and is subject to transcriptional inhibition by JAZs. We speculate that viral infection induces an increase in JA levels, and upon JA signal perception, COI1s recruit JAZs for degradation, thereby releasing OsMYB4P to activate the expression of genes essential for JA‐mediated antiviral defence in rice (Figure 7h).

Most JA‐responsive MYB TFs reported so far belong to the R2R3‐type MYB family. For instance, *in Arabidopsis*, MYB21, MYB24 and MYB75 are well‐studied JA‐responsive TFs belonging to the R2R3‐type MYB family (Huang *et al*., 2017; Qi *et al*., 2011; Song *et al*., 2011). A common feature of these TFs is their interaction with JAZ proteins, which in turn suppress the transcriptional activity of MYB21, MYB24 and MYB75. Upon sensing the JA signal, COI1 recruits JAZs to the SCF^COI1^ complex for ubiquitination and degradation, thereby releasing MYB21, MYB24 and MYB75 to activate the expression of genes essential for JA‐mediated processes such as anther development, filament elongation, and trichome initiation in *Arabidopsis* (Huang *et al*., 2017; Qi *et al*., 2011; Song *et al*., 2011). Consistent with these findings, Co‐IP and BiFC assays confirmed that OsMYB4P interacts with OsJAZ9 and OsJAZ11 (Figure 5a–c). Interestingly, similar to JAMYB, no interaction between JAMYB and JAZs was observed in the Y2H system, while in the plant system, JAMYB was able to interact with JAZ6 (Yang *et al*., 2020). Furthermore, OsJAZ9 and OsJAZ11 also suppress the transcriptional activity of OsMYB4P, as shown by dual‐luciferase transient transcriptional activity assays (Figure 5d,e). Moreover, MeJA treatment rapidly induced *OsMYB4P* expression within a short time (Figure S4c). These results identify OsMYB4P as a JA‐responsive TF. Furthermore, evaluations of JA content and sensitivity revealed that OsMYB4P positively regulates the JA pathway (Figure 4d–f; Figure S4b). On the other hand, MYB21, MYB24 and MYB75 primarily participate in regulating JA‐mediated plant growth and development. Although the role of the JA pathway in plant antiviral defence is well‐documented, studies focusing on the involvement of R2R3‐type MYB TFs in JA‐mediated antiviral defence remain limited. To address whether OsMYB4P contributes to rice antiviral defence, we inoculated *OsMYB4P* knockout and overexpressing transgenic plants with SRBSDV and RSV. Our results revealed that OsMYB4P mediates broad‐spectrum antiviral defence against both SRBSDV and RSV infection in rice (Figure 2). Interestingly, this role is similar to the previously characterized JA‐responsive TF JAMYB, another member of the R2R3‐type MYB family. JAMYB has been reported to bind to the *AGO18* promoter, activating *AGO18* expression and enhancing antiviral RNA silencing pathways, thereby promoting the rice antiviral immune response to RSV infection (Yang *et al*., 2020).

The homologue of OsMYB4P in *Arabidopsis*, AtMYB60, has been previously reported as a negative regulator of oxylipin synthesis in stomata by negatively regulating the expression of *13‐LOXs* (*LOX2*, *LOX4 and LOX6*) (Abdullah‐Zawawi *et al*., 2021; Simeoni *et al*., 2022). In contrast, our findings reveal that OsMYB4P acts as a transcriptional activator, promoting the expression of *CM‐LOX1* (Figure 4b,c; Figure S4a). This is consistent with a previous report demonstrating that OsMYB4P functions as a transcriptional activator to regulate the expression of Pi homeostasis‐related genes, thereby increasing phosphate acquisition in rice (Yang *et al*., 2014). The functional divergence between homologous proteins prompted us to conduct an amino acid homology analysis. The results showed that OsMYB4P and AtMYB60 share high homology in the N‐terminal MYB DNA binding domain (DBD), while their C‐terminal sequences exhibit significant divergence (Figure S10). It is well‐established that R2R3‐type MYB TFs not only possess a conserved DBD but also include regions of variable length and low sequence conservation (Dubos *et al*., 2010; Millard *et al*., 2019; Stracke *et al*., 2001), commonly referred to as ‘non‐MYB regions’. Despite their high divergence, these non‐MYB regions of plant R2R3‐type MYB TFs retain essential molecular functions critical to the TF's overall activity, such as harbouring activation and repression domains (Millard *et al*., 2019). Thus, the low sequence conservation within the non‐MYB regions of OsMYB4P and AtMYB60 likely accounts for their functional differences in transcriptional regulation. In addition, upon RSV infection, JA signalling was significantly activated in *OE‐OsMYB4P* lines compared to the NIP plants (Figure 3). Notably, JA‐mediated defence‐related genes, such as *PR1a*, *PR2 and PR5* showed markedly increased expression (Figure 3d). Collectively, these findings further highlight the role of OsMYB4P as a transcriptional activator in modulating JA‐responsive antiviral defence in rice.

In the arms race between viruses and hosts, viruses encode proteins that employ various strategies to suppress or interfere with the host's JA‐mediated antiviral defences in order to facilitate infection (Li *et al*., 2024). For example, SP8 and P2 act as viral transcriptional repressors, both targeting the JA master transcription factors OsMYC2 and OsMYC3, leading to severe repression of the JA cascade that benefits viral pathogenicity and vector transmission (Li *et al*., 2021). RSV P2 disrupts the interaction between OsHDA706 and OsLOX14, reducing the lysine deacetylation and protein accumulation levels of OsLOX14, thereby manipulating the JA biosynthesis pathway to promote viral infection (Yang *et al*., 2024). JA signalling serves as a central hub in the multilayered regulation of rice immunity, with a strong ability to modulate downstream antiviral gene expression. Thus, suppressing JA‐mediated defence is a common broad‐spectrum strategy among diverse viruses (Li *et al*., 2024). In our study, we also found that both SP8 and P2 interact with OsMYB4P (Figure 1). Similar to their interaction with OsMYC2, SP8 and P2 function as transcriptional repressors, significantly inhibiting OsMYB4P's transcriptional activity (Figure 5f). Furthermore, OsJAZs also interact with SP8 and P2, further enhancing their viral transcriptional repressor activity (Li *et al*., 2021) (Figure 5g). These findings indicate that the JA‐responsive OsMYB4P serves as a potential broad‐spectrum antiviral target. According to published studies on the interactions between TFs and viral proteins, it has been observed that viral proteins often inhibit the oligomerization of TFs, which is critical for their transcriptional activity or DNA binding ability. For example, SP8 modulates auxin signalling by binding to the CTD domain of OsARF17, thereby inhibiting its transcriptional activation and disrupting its dimerization (Zhang *et al*., 2020). Similarly, tomato spotted wilt virus (TSWV)‐encoded NSs protein disrupts the dimerization of TCP17, thereby inhibiting its transcriptional activation and further impairing auxin response signalling, which ultimately facilitates TSWV infection (Zhao *et al*., 2024). Consistent with these findings, OsMYB4P forms higher‐order oligomers *in planta* (Figure 6a,b; Figure S8a,b). SP8 and P2, in addition to directly affecting OsMYB4P's transcriptional activity, also inhibit its oligomer formation, further weakening its transcriptional activity and DNA binding ability, thereby suppressing JA‐mediated expression of defence‐related genes (Figures 6c–f, 7a). Additionally, we observed a significant reduction in OsMYB4P protein levels in the presence of viral proteins SP8 and P2, with no corresponding change in transcript levels, indicating that SP8 and P2 significantly destabilize OsMYB4P protein (Figure 7b–e). As dimers or oligomers are generally more stable than monomers (Messaritou *et al*., 2010; Neet and Timm, 1994; Sinha and Surolia, 2005), the disruption of OsMYB4P self‐interaction by viral proteins likely induces its dissociation into unstable monomers. OsMYB4P is ubiquitinated, and its degradation is suppressed by MG132, suggesting that OsMYB4P is degraded via the 26S proteasome pathway (Figure 7). Together, our study identifies OsMYB4P as a novel JA‐responsive R2R3‐type transcription factor crucial for JA‐mediated broad‐spectrum antiviral defence against SRBSDV and RSV (Figure 7h).

## Methods

### Plant material and growth conditions

Transgenic rice plants overexpressing *OsMYB4P* were generated using *Nipponbare* (NIP) as the genetic background, while mutant plants were produced in the *Zhonghua 11* (ZH11) background. The *OsMYB4P* coding sequence was cloned into the *pCAMBIA1300* vector with an MYC tag and introduced into rice through *Agrobacterium tumefaciens* (*A. tumefaciens*)‐mediated transformation (Wimi Biotechnology, Hainan, China). High expression lines were selected by quantitative reverse transcription PCR (qRT‐PCR) to evaluate *OsMYB4p* expression levels in the overexpression (OE) lines. The mutant plants were bought from Biogle Genetech (www.biogle.cn). DNA sequencing was performed to confirm lines with premature termination of protein translation. Rice plants were cultivated in a greenhouse maintained at 28–30 °C with a 14/10 h light/dark cycle, while *N. benthamiana* plants were grown at 25°C under a 16/8 h light/dark cycle. All primers used for constructing mutant rice lines and vectors are listed in Table S1.

### Virus inoculation assay

The rice virus inoculation methods have been previously described in detail (Zhang *et al*., 2020). For SRBSDV inoculation, the newly hatched instar nymphs of *Sogatella furcifera* (*Horváth*) were fed on SRBSDV‐infected rice plants for 3–4 days, followed by a 10‐day virulence induction period. Subsequently, third‐instar infected WBPH (*white‐backed planthopper*) nymphs were introduced to rice plants at the four‐leaf stage at a ratio of 1:2 (nymphs:plants) and allowed to feed for 3 days. The SRBSDV‐inoculated plants were then grown in the field for approximately 30 days, after which the degree of viral infection was evaluated using qRT‐PCR and Western blotting. For RSV inoculation, the host insect was *Laodelphax striatellus* (*Fallén*). The methods used were similar to those described for SRBSDV inoculation. The RSV‐inoculated plants were then grown in the field for approximately 20 days. All experiments were repeated at least three times with similar results. The specific primers used for detecting SRBSDV/RSV are listed in Table S1.

### Yeast two‐hybrid assay

For Y2H assay, the full‐length coding sequences were amplified by PCR and then cloned into the *pGBKT7* vector containing the DNA binding domain (BD) as bait and the *pGADT7* vector containing the activation domain (AD). The constructed *pGADT7* vectors and *pGBKT7* vectors were co‐transformed into the yeast AH109 (Biyotime) following previously described methods (Zhang *et al*., 2020). The transformed yeast cells were cultured on SD/−Leu/−Trp medium (lacking leucine and tryptophan) for 3 days, and then positive clones were transferred to SD/−Leu/−Trp/−His/−Ade medium (lacking adenine, histidine, leucine and tryptophan) for selection and interaction tests. All yeast cultures were maintained at 30 °C.

### Bimolecular fluorescence complementation and subcellular localization assays

The full‐length coding sequences of the target genes were cloned into the *PCV‐nYFP* (N terminus of YFP) vector and the *PCV‐cYFP* (C terminus of YFP) vector. These constructs were introduced into *A. tumefaciens* strain GV3101 using electroporation. Different combinations of constructs were co‐expressed in *N. benthamiana* leaves for 48 h. YFP fluorescence was detected using a laser confocal microscope (Leica SP8X). For the subcellular localization assay, the full‐length coding sequence of *OsMYB4P* was cloned and inserted into the *pCAMBIA1300* vector tagged with GFP. Then, the constructed vector was transformed into *A. tumefaciens* strain GV3101 and expressed in *N. benthamiana* leaves for 48 h. Laser confocal microscopy was used to detect GFP fluorescence signals. All experiments were repeated three times with similar results.

### Co‐immunoprecipitation and competitive Co‐IP assay

The full‐length coding sequences of the target proteins were cloned into the *pCAMBIA1300* vector tagged with GFP, FLAG and MYC. GUS‐MYC, GFP‐FLAG and HA‐GFP were used as negative controls. These constructs were introduced into *A. tumefaciens* strain GV3101 using electroporation. Different combinations of constructs were co‐expressed in *N. benthamiana* leaves for 48 h. Then, leaves were harvested and ground into a fine powder using liquid nitrogen. Total proteins were extracted with IP buffer (40 mM Tris–HCl pH 7.5, 100 mM NaCl, 4 mM MgCl_2_, 1 mM EDTA, 1 mM DTT, 1 mM PMSF, 1% glycerol, 0.2% Triton X‐100, 50 μM MG132, 2× Protease inhibitor cocktail and ddH_2_O) on ice for 10 min. The extracts were centrifuged twice at 15387 ***g*** for 10 min at 4 °C and the supernatant was collected. The supernatant was then immunoprecipitated using the appropriate magnetic beads to enrich the tagged proteins at 4 °C for 1.5 h. After incubation, the supernatant was removed, and the magnetic beads bound to the target protein were washed three times with IP buffer and SDS‐PAGE gels were used to isolate proteins.

For the competitive Co‐IP assay, OsMYB4P‐FLAG, OsMYB4P‐GFP and SP8/P2‐MYC constructs were co‐expressed in *N. benthamiana* leaves. GUS‐MYC was used as the negative control. After 24 h of infiltration, MG132 (Sigma, Saint Louis, USA) was added at a final concentration of 50 μM to prevent SP8/P2‐induced degradation of OsMYB4P. After 48 h of infiltration, the tobacco leaves were harvested for Co‐IP assays.

### Plant protein extraction and Western blotting

The rice and *N. benthamiana* plant samples were ground into a powder in liquid nitrogen, and then 2× SDS loading buffer (100 mM Tris–HCl pH 6.8, 4% SDS, 0.2% BPB, 20% Glycerol and 2% β‐mercaptoethanol) was added for lysis on ice for 10 min. The samples were then boiled in hot water for 5 min. The total protein extracts were centrifuged at 12 000 ***g*** for 10 min and the supernatant was separated on SDS‐PAGE gels. For the analysis of native protein complexes, samples were lysed using IP buffer and then mixed with loading buffer (200 mM Tris–HCl, pH 6.8, 0.01% bromophenol blue and 40% glycerol). Without boiling, the proteins were separated on a non‐denaturing precast protein plus gel (Yeasen Biotechnology, Shanghai, China). Electrophoresis was performed in a native PAGE running buffer at 40 V. The isolated proteins were transferred from the gel to the PVDF membrane, and the corresponding antibodies were used for protein detection: Anti‐MYC antibody (1:5000 dilution, TransGen Biotech, Beijing, China), Anti‐FLAG antibody (1:5000 dilution, TransGen Biotech), Anti‐GFP antibody (1:5000 dilution, TransGen Biotech), Anti‐RSV‐CP, Anti‐SRBSDV‐P10, Anti‐His antibody (1:5000 dilution, TransGen Biotech), Anti‐Ubiquitin (P4D1) antibody (3:5000 dilution, Santa Cruz Biotechnology, Dallas, USA), Goat anti‐mouse (1:10 000 dilution, TransGen Biotech) and Goat anti‐rabbit (1:10000 dilution, TransGen Biotech).

### Total RNA extraction and qRT‐PCR analyses

Total RNA was extracted from rice and *N. benthamiana* using TRIzol Reagent (Thermo Fisher Scientific, Waltham, USA), following the method described previously. 1000 ng total RNA was reverse‐transcribed into first strand cDNA using the Hiscript II RT SuperMix (Vazyme, Nanjing, China). The obtained cDNA served as the template for the quantitative real‐time PCR (qRT‐PCR) reactions using ChamQ SYBR qPCR Master Mix (Without Rox, Vazyme). The qRT‐PCR reactions were conducted on an ABI QuantStudio 5 system (Thermo Fisher Scientific), and relative expression levels were calculated using the 2^−ΔΔCt^ method. *OsUBQ5* was used as the internal parameter in rice, and *actin* was used as the internal control for tobacco. All primers used for qRT‐PCR analyses are listed in Table S1.

### *In vivo* protein degradation and ubiquitination assays

For the protein degradation assays, OsMYB4P‐GFP was co‐expressed with SP8‐MYC, P2‐MYC or GUS‐MYC (as a control) in *N. benthamiana* leaves. At 24 h post‐infiltration, the leaves were treated with either 50 μM MG132 (Sigma) or DMSO. At 48 h post‐infiltration, samples were collected to estimate OsMYB4P protein levels by Western blot using an anti‐GFP antibody. RbcL served as a loading control. For the ubiquitination assays, OsMYB4P‐FLAG was co‐expressed with either HA‐GFP (as a control), P2‐GFP or SP8‐GFP in *N. benthamiana* leaves. At 48 h post‐infiltration, leaf samples were lysed in IP buffer for 10 min at 4 °C, followed by immunoprecipitation using appropriate magnetic beads at 4 °C for 3.5 h to enrich tagged proteins. The beads were then washed with 1× PBS and boiled for 5 min. Proteins were separated by SDS‐PAGE and analysed using anti‐ubiquitin antibodies to detect OsMYB4P ubiquitination.

### Dual‐luciferase transient transcriptional activity assay

The dual‐luciferase reporter system was performed as previously described (Li *et al*., 2021). Briefly, a 2.0‐kb promoter fragment of *CM‐LOX1* (LOC_Os08g39840) was amplified and cloned into the *pGreenII0800‐LUC* vector to generate reporter constructs *pOsCM‐LOX1::LUC*. The coding sequences of *OsMYB4P*, *OsJAZ9*, *OsJAZ11*, *SP8* and *P2* were inserted into the *pCAMBIA1300* vector to generate effector constructs. These constructs were transformed into *A. tumefaciens* strain GV3101 as described in the BiFC assay. The indicated combinations of constructs, as shown in Figures 4 and 5, were co‐expressed in *N. benthamiana* leaves for 48 h. The expression levels of firefly and Renilla luciferases in *N. benthamiana* leaves were measured using the Dualucif® Firefly & Renilla Assay Kit (UElandy, Suzhou, China) and a microplate reader (BioTek Synergy H1).

### Electrophoretic mobility shift assay

For protein purification, the full‐length coding sequences of the *OsMYB4P*, *SP8* and *P2* were cloned into the *pET‐32a* (His tag) vector or *pGEX‐4T‐2* (GST tag) vector and expressed as tag fusion proteins (His‐OsMYB4P, GST‐SP8 and GST‐P2, respectively) in *Escherichia coli* BL21‐Star (DE3) pLysS cells. His‐OsMYB4P was purified using a HisTrap™ HP column (GE Healthcare, Chicago, USA, Cat#: 17524802), while GST‐SP8 and GST‐P2 were purified using a GSTrap™ HP column (GE Healthcare, Cat#: 17528202). Promoter fragments from *CM‐LOX1* containing the ACCGGT motif were synthesized (Sangon Biotech, Shanghai, China) and labelled with biotin at their 5′ end as the hot probes, with unlabeled used as competition probes. For EMSA, probes were incubated with recombinant proteins in the indicated combinations (as shown in Figures 4c and 7a), at 25 °C in a reaction system prepared according to the manufacturer's instructions for the LightShift™ EMSA Optimization & Control Kit (Thermo Fisher Scientific). The free and bound probes were separated on 4–6% native PAGE gels (5× TBE, 30% Acr‐Bis, 40% glycerinum, 10% APS, TEMED and ddH_2_O), and the shifted signals were visualized using the Chemiluminescent Nucleic Acid Detection Module with the BIO‐RAD ChemiDoc MP Imaging System.

### JA sensitivity, content, and response assays

For JA sensitivity assays, seeds of transgenic and control rice plants were germinated at 37°C. Seeds with uniform root length and similar growth were selected and transferred into rice nutrient solution in a hydroponic box containing 0, 0.5 and 1.0 μM MeJA. Plants were grown under an 8/16‐h light/dark cycle at 30°C for 5 days. The primary root lengths were measured and recorded, and relative root length was calculated to evaluate the sensitivity to JA. For JA content assays, 10‐day‐old transgenic and control rice plants were collected and JA levels were measured according to previously described methods (Yang *et al*., 2024). For JA response assays, 10‐day‐old transgenic and control rice plants were sprayed with MeJA (50 μM), and samples were collected at 0 h and 0.5 h. To analyse the response of *OsMYB4P* to JA treatment, wild‐type (NIP) plants were sprayed with 50 μM MeJA and samples were collected at 0, 10, 15 and 20 min. Total RNA was extracted from the samples, and qRT‐PCR was performed to analyse the expression of JA signal‐related genes.

### RNA‐Seq analysis

RNA library construction and RNA sequencing were performed as described previously (Wu *et al*., 2024). Rice leaves from mock and RSV‐infected plants were collected at 20 days' post‐infection (dpi), ground into powder in liquid nitrogen, and then subjected to total RNA extraction. The RNA quantity assessment and sequencing were carried out by Seqhealth Technology Co., Ltd. (Wuhan, China). Briefly, total RNA was used as input to prepare RNA sequencing libraries using the KC™ Digital mRNA Library Prep Kit (Seqhealth Tech. Co., Ltd., Wuhan, China) following the manufacturer's instructions. The libraries were then quantified and sequenced using the PE150 sequencing mode on the DNBSEQ‐T7 platform (MGI). The RNA‐seq data have been deposited in the Genome Sequence Archive (GSA) at the National Genomics Data Center, part of the China National Center for Bioinformation/Beijing Institute of Genomics, Chinese Academy of Sciences, under the BioProject accession number CRA024685.

## Conflict of interest

The authors have not declared a conflict of interest.

## Supporting information


**Figure S1** Construction and validation of *OsMYB4P* knockout and overexpression mutants. (a) Construction of *osmyb4p* knockout (CRISPR‐Cas9) transgenic rice plants. The sgRNA sequence targeting *OsMYB4P* is indicated. The mutations include an ‘A’ insertion in line *osmyb4p#1* and a ‘T’ insertion in line *osmyb4p#3*, both resulting in premature translational termination of *OsMYB4P*. (b) qRT‐PCR analysis of *OsMYB4P* transcript levels in 12‐day‐old *OE‐OsMYB4P* plants. Data are shown as means ± SD of three biological replicates. Asterisks indicate significant differences. **P < 0.01 and ***P < 0.001 (Student's *t* test).
**Figure S2** Evaluation of the transcriptome in response to RSV infection. (a) Principal component analysis (PCA) of the transcriptomic profiling data obtained from RNA‐Seq. (b) Hierarchical clustering analysis of the expression of JA biosynthetic genes, including *CM‐LOX1*, *OsOPR4*, *OsOPR5* and *LOC_Os03g53010*.
**Figure S3** Subcellular localization of OsMYB4P and analysis of its potential binding motif. (a) Subcellular localization analysis shows that OsMYB4P, fused with GFP, is localized in the nucleus. The histone H2B serves as a nucleus marker. Scale bar, 50 μm. (b) The potential binding motif ACCGGT of OsMYB4P was identified using the online tool PlantPAN4.0 (Chow *et al*., 2024).
**Figure S4** Analysis of *CM‐LOX1* expression, JA content in *OE‐OsMYB4P* plants and JA‐induced *OsMYB4P* expression. (a) qRT‐PCR analysis showing significantly higher *CM‐LOX1* expression in *OE‐OsMYB4P* plants. (b) JA content analysis indicating a significant increase in JA levels in *OE‐OsMYB4P* plants. (c) qRT‐PCR analysis showing rapid induction of *OsMYB4P* expression in response to 50 μM MeJA treatment. Data are shown as means ± SD of three biological replicates. Asterisks indicate significant differences. *P < 0.05, **P < 0.01 and ***P < 0.001 (Student's *t* test).
**Figure S5** Analysis of the interactions between OsJAZs and OsMYB4P by Y2H assays. (a) Analysis of the interactions between OsJAZs and full‐length OsMYB4P. (b) Analysis of the interactions between OsJAZs and the truncated version of OsMYB4P (OsDBD). (c) Analysis of the interactions between OsJAZs and the truncated version of OsMYB4P (OsCTD).
**Figure S6** Analysis of the interactions between OsJAZs and OsMYB4P by BiFC and Co‐IP assays. (a) Analysis of the interactions between OsJAZs and OsMYB4P by BiFC assays. BiFC assays indicate that OsMYB4P interacts specifically with OsJAZ4 and OsJAZ6 *in planta*. Scale bar, 50 μm. (b) Analysis of the interactions between OsJAZ4 and OsMYB4P by Co‐IP assays. OsMYB4P‐FLAG was co‐expressed with the OsJAZ4‐MYC or GUS‐MYC (negative control) in *N. benthamiana* leaves. (c) Analysis of the interactions between OsJAZ6 and OsMYB4P by Co‐IP assays. OsMYB4P‐FLAG was co‐expressed with the OsJAZ6‐MYC or GUS‐MYC (negative control) in *N. benthamiana* leaves. The red asterisks point to the specific band. (d) Schematic diagram of the multiple effectors and reporter used in the dual‐luciferase transient transcriptional activity assay. The reporter gene *LUC* was driven by the *CM‐LOX1* promoter, while the effectors SP8, P2, OsJAZ9, OsJAZ11 and OsMYB4P were driven by the *CaMV 35S* promoter.
**Figure S7** Self‐interaction analysis of OsMYB4P, OsDBD and OsCTD in the Y2H system. (a) Y2H assays indicate that OsMYB4P and OsCTD exhibit self‐activation in the Y2H system, making this system unsuitable for detecting the self‐interaction of OsMYB4P and OsCTD. However, BK‐OsDBD does not interact with AD‐OsDBD or AD‐OsCTD in the Y2H system. (b) Co‐IP assays show that the OsDBD interacts with OsCTD. OsCTD‐FLAG was co‐expressed with the OsDBD‐GFP or HA‐GFP (negative control) in *N. benthamiana* leaves.
**Figure S8** Native‐PAGE analysis shows that OsMYB4P forms an oligomer. (a) OsMYB4P‐FLAG was expressed in *N. benthamiana* leaves, and total proteins were extracted under denaturing and non‐denaturing conditions. Western blot analysis with anti‐FLAG antibodies showed a ~ 50 kDa band under denaturing conditions and a ~ 250 kDa band in native‐PAGE. (b) Purified His‐tagged OsMYB4P analysed with anti‐His antibodies yielded similar results.
**Figure S9** qRT‐PCR analysis of *OsMYB4P* transcript levels in the leaves from Figure 7b,c.
**Figure S10** Similarity analysis of orthologs of OsMYB4P and AtMYB60. Sequence alignment of rice OsMYB4P and *Arabidopsis* AtMYB60 was performed using the ClustalW program (https://www.genome.jp/tools‐bin/clustalw) and ESPript 3.0 tool (https://espript.ibcp.fr/ESPript/cgi‐bin/ESPript.cgi).

**Table S1** Primers used in this study.

**Table S2** The differentially expressed genes in OE‐MYB4P_M versus NIP_M, OE‐MYB4P_R versus NIP_R and NIP_R versus NIP_M subsets.

## Data Availability

The data that support the findings of this study are openly available in Genome Sequence Archive (GSA) at https://ngdc.cncb.ac.cn/gsa, reference number CRA024685.
