# Supplementary material for: JA‐responsive R2R3‐type MYB transcription factor OsMYB4P confers broad‐spectrum antiviral immunity in rice
Source: Plant Biotechnol J. 2025 Jul 13;23(10):4602–17. doi: 10.1111/pbi.70246 (PMC12483970; doi:10.1111/pbi.70246)
Supplement: Supplementary file 1 — Figure S1 Construction and validation of OsMYB4P knockout and overexpression mutants. (a) Construction of osmyb4p knockout (CRISPR‐Cas9) transgenic rice plants. The sgRNA sequence targeting OsMYB4P is indicated. The mutations include an ‘A’ insertion in line osmyb4p#1 and a ‘T’ insertion in line osmyb4p#3, both resulting in premature translational termination of OsMYB4P. (b) qRT‐PCR analysis of OsMYB4P transcript levels in 12‐day‐old OE‐OsMYB4P plants. Data are shown as means ± SD of three biological replicates. Asterisks indicate significant differences. **P < 0.01 and ***P < 0.001 (Student's t test). Figure S2 Evaluation of the transcriptome in response to RSV infection. (a) Principal component analysis (PCA) of the transcriptomic profiling data obtained from RNA‐Seq. (b) Hierarchical clustering analysis of the expression of JA biosynthetic genes, including CM‐LOX1, OsOPR4, OsOPR5 and LOC_Os03g53010. Figure S3 Subcellular localization of OsMYB4P and analysis of its potential binding motif. (a) Subcellular localization analysis shows that OsMYB4P, fused with GFP, is localized in the nucleus. The histone H2B serves as a nucleus marker. Scale bar, 50 μm. (b) The potential binding motif ACCGGT of OsMYB4P was identified using the online tool PlantPAN4.0 (Chow et al., 2024). Figure S4 Analysis of CM‐LOX1 expression, JA content in OE‐OsMYB4P plants and JA‐induced OsMYB4P expression. (a) qRT‐PCR analysis showing significantly higher CM‐LOX1 expression in OE‐OsMYB4P plants. (b) JA content analysis indicating a significant increase in JA levels in OE‐OsMYB4P plants. (c) qRT‐PCR analysis showing rapid induction of OsMYB4P expression in response to 50 μM MeJA treatment. Data are shown as means ± SD of three biological replicates. Asterisks indicate significant differences. *P < 0.05, **P < 0.01 and ***P < 0.001 (Student's t test). Figure S5 Analysis of the interactions between OsJAZs and OsMYB4P by Y2H assays. (a) Analysis of the interactions between OsJAZs and full‐length Os [file PBI-23-4602-s002.pdf]

Figure S1

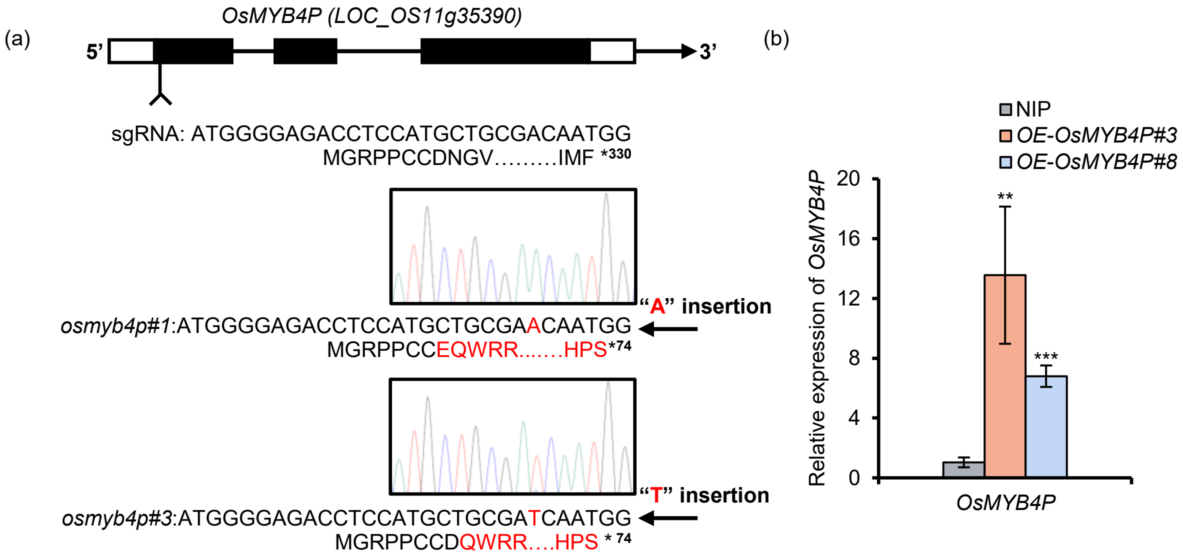
(a)
OsMYB4P (LOC_OS11g35390)
5'
3'
sgRNA: ATGGGGAGACCTCCATGCTGCGACAATGG
MGRPPCCDNGV.........IMF *330
"A" insertion
osmyb4p#1:ATGGGGAGACCTCCATGCTGCGAACAATGG
MGRPPCCEQWRR.......HPS*74
"T" insertion
osmyb4p#3:ATGGGGAGACCTCCATGCTGCGATCAATGG
MGRPPCCDQWRR....HPS * 74
(b)
NIP
OE-OsMYB4P#3
OE-OsMYB4P#8
Relative expression of OsMYB4P
20
16
12
8
4
0
**
***
OsMYB4P

Figure S2

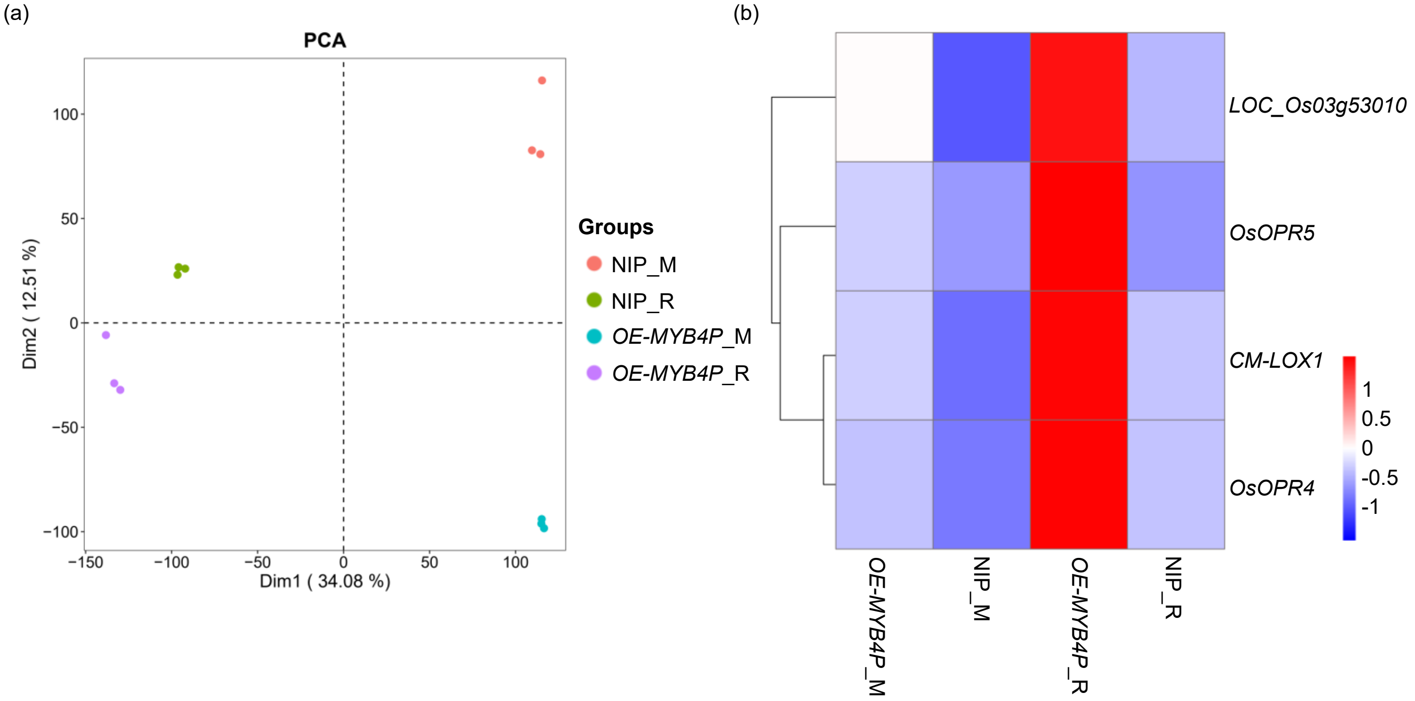
(a)
PCA
Dim2 (12.51 %)
Dim1 (34.08 %)
100
50
0
−50
−100
−150
−100
−50
0
50
100
Groups
NIP_M
NIP_R
OE-MYB4P_M
OE-MYB4P_R
(b)
LOC_Os03g53010
OsOPR5
CM-LOX1
OsOPR4
1
0.5
0
-0.5
-1
OE-MYB4P_M
NIP_M
OE-MYB4P_R
NIP_R

Figure S3

(a)

(b)

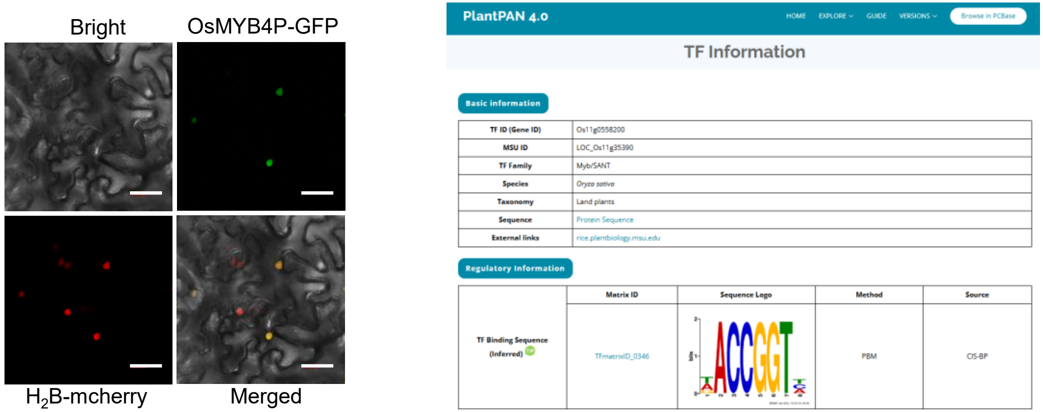
Bright
OsMYB4P-GFP
H2B-mcherry
Merged
PlantPAN 4.0
HOME
EXPLORE
GUIDE
VERSIONS
Browse in PCBase
TF Information
Basic information
TF ID (Gene ID)
Os11g0558200
MSU ID
LOC_Os11g35390
TF Family
Myb/SANT
Species
Oryza sativa
Taxonomy
Land plants
Sequence
Protein Sequence
External links
rice.plantbiology.msu.edu
Regulatory Information
Matrix ID
Sequence Logo
Method
Source
TF Binding Sequence (Inferred)
TFmatrixID_0346
PBM
CIS-BP

Figure S4

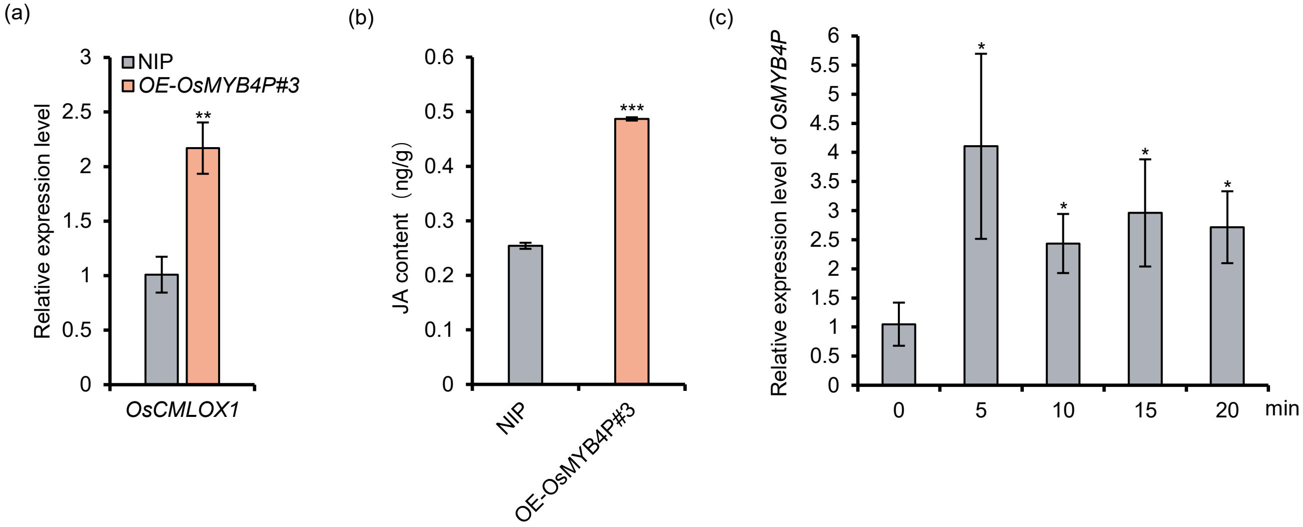

(a)
NIP
OE-OsMYB4P#3
Relative expression level
**
OsCMLOX1
(b)
JA content（ng/g）
***
NIP
OE-OsMYB4P#3
(c)
Relative expression level of OsMYB4P
*
0
5
10
15
20
min

Figure S5

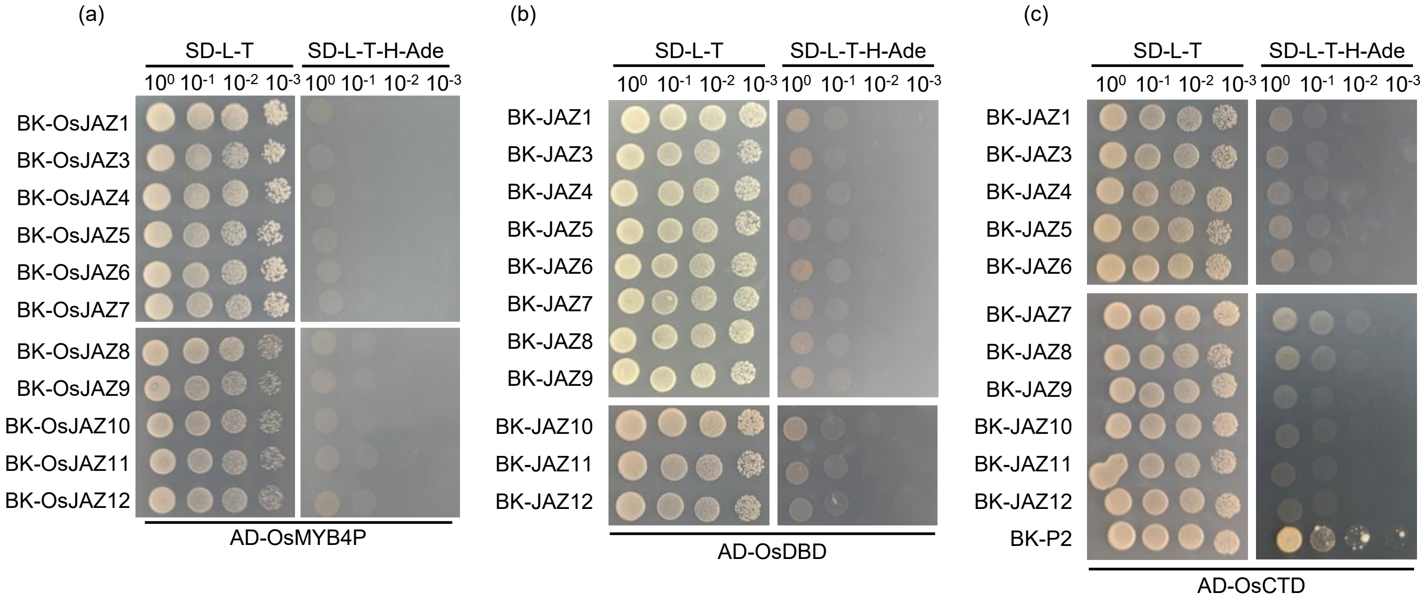

(a)
SD-L-T
SD-L-T-H-Ade
10^0 10^-1 10^-2 10^-3 10^0 10^-1 10^-2 10^-3
BK-OsJAZ1
BK-OsJAZ3
BK-OsJAZ4
BK-OsJAZ5
BK-OsJAZ6
BK-OsJAZ7
BK-OsJAZ8
BK-OsJAZ9
BK-OsJAZ10
BK-OsJAZ11
BK-OsJAZ12
AD-OsMYB4P
(b)
SD-L-T
SD-L-T-H-Ade
10^0 10^-1 10^-2 10^-3 10^0 10^-1 10^-2 10^-3
BK-JAZ1
BK-JAZ3
BK-JAZ4
BK-JAZ5
BK-JAZ6
BK-JAZ7
BK-JAZ8
BK-JAZ9
BK-JAZ10
BK-JAZ11
BK-JAZ12
AD-OsDBD
(c)
SD-L-T
SD-L-T-H-Ade
10^0 10^-1 10^-2 10^-3 10^0 10^-1 10^-2 10^-3
BK-JAZ1
BK-JAZ3
BK-JAZ4
BK-JAZ5
BK-JAZ6
BK-JAZ7
BK-JAZ8
BK-JAZ9
BK-JAZ10
BK-JAZ11
BK-JAZ12
BK-P2
AD-OsCTD

Figure S6

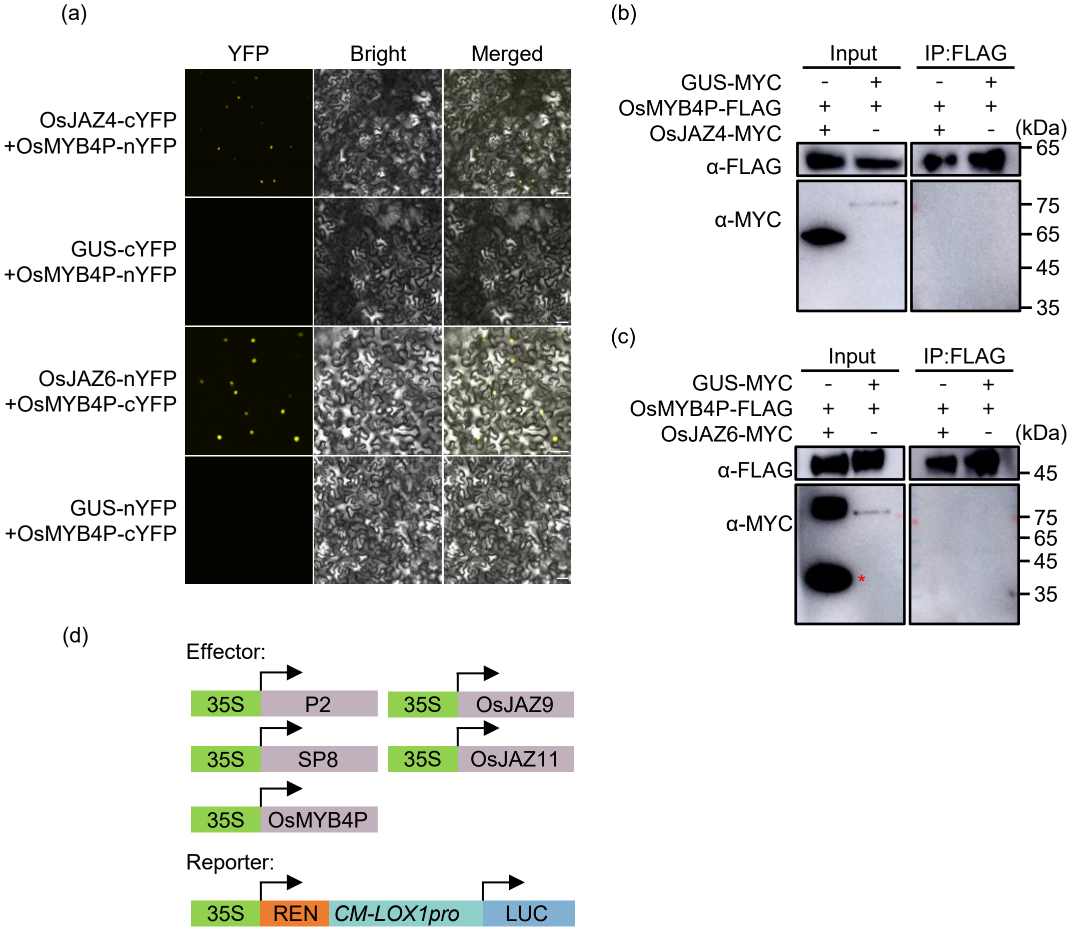

(a)
YFP
Bright
Merged
OsJAZ4-cYFP
+OsMYB4P-nYFP
GUS-cYFP
+OsMYB4P-nYFP
OsJAZ6-nYFP
+OsMYB4P-cYFP
GUS-nYFP
+OsMYB4P-cYFP
(b)
Input
IP:FLAG
GUS-MYC - + - +
OsMYB4P-FLAG + + + +
OsJAZ4-MYC + - + -
(kDa)
α-FLAG
α-MYC
65
75
65
45
35
(c)
Input
IP:FLAG
GUS-MYC - + - +
OsMYB4P-FLAG + + + +
OsJAZ6-MYC + - + -
(kDa)
α-FLAG
α-MYC
45
75
65
45
35
*
(d)
Effector:
35S
P2
35S
OsJAZ9
35S
SP8
35S
OsJAZ11
35S
OsMYB4P
Reporter:
35S
REN
CM-LOX1pro
LUC

Figure S7

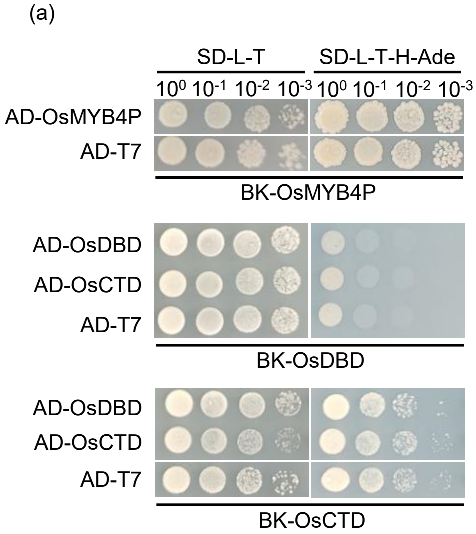

(a)
SD-L-T
SD-L-T-H-Ade
10^0 10^-1 10^-2 10^-3 10^0 10^-1 10^-2 10^-3
AD-OsMYB4P
AD-T7
BK-OsMYB4P
AD-OsDBD
AD-OsCTD
AD-T7
BK-OsDBD
AD-OsDBD
AD-OsCTD
AD-T7
BK-OsCTD

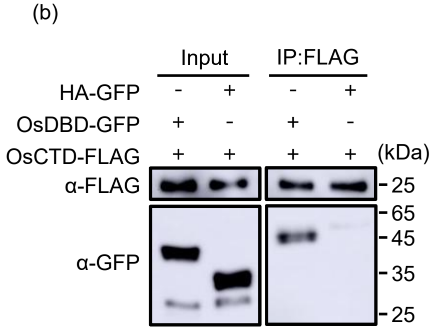

(b)
Input
IP:FLAG
HA-GFP - + - +
OsDBD-GFP + - + -
OsCTD-FLAG + + + +
(kDa)
α-FLAG
25
α-GFP
65
45
35
25

Figure S8

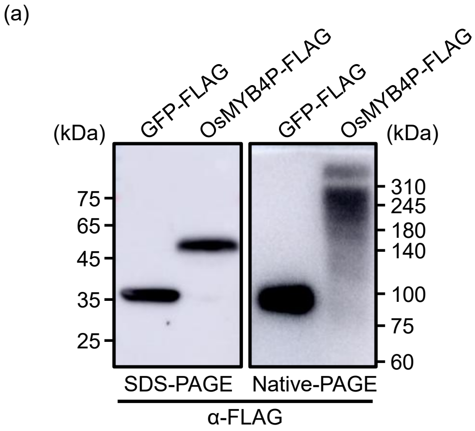
(a)
GFP-FLAG
OsMYB4P-FLAG
GFP-FLAG
OsMYB4P-FLAG
(kDa)
75
65
45
35
25
(kDa)
310
245
180
140
100
75
60
SDS-PAGE
Native-PAGE
α-FLAG

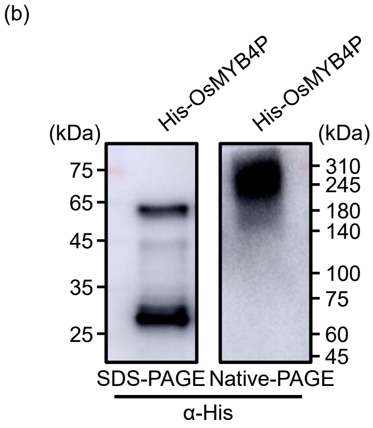
(b)
His-OsMYB4P
His-OsMYB4P
(kDa)
75
65
45
35
25
(kDa)
310
245
180
140
100
75
60
45
SDS-PAGE
Native-PAGE
α-His

Figure S9

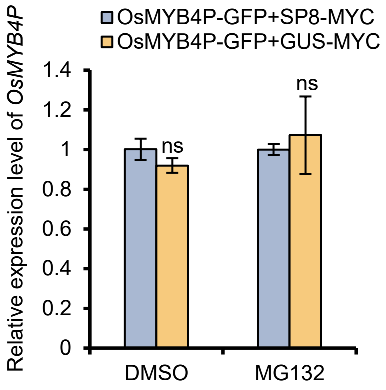

OsMYB4P-GFP+SP8-MYC
OsMYB4P-GFP+GUS-MYC
Relative expression level of OsMYB4P
1.4
1.2
1
0.8
0.6
0.4
0.2
0
ns
ns
DMSO
MG132

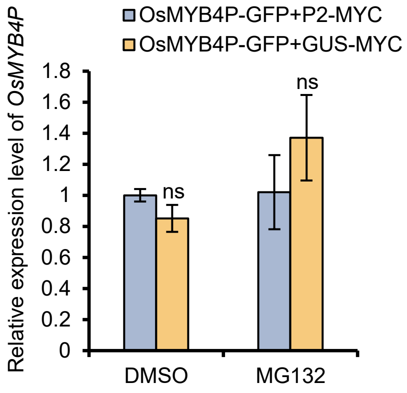

OsMYB4P-GFP+P2-MYC
OsMYB4P-GFP+GUS-MYC
Relative expression level of OsMYB4P
1.8
1.6
1.4
1.2
1
0.8
0.6
0.4
0.2
0
ns
ns
DMSO
MG132

Figure S10

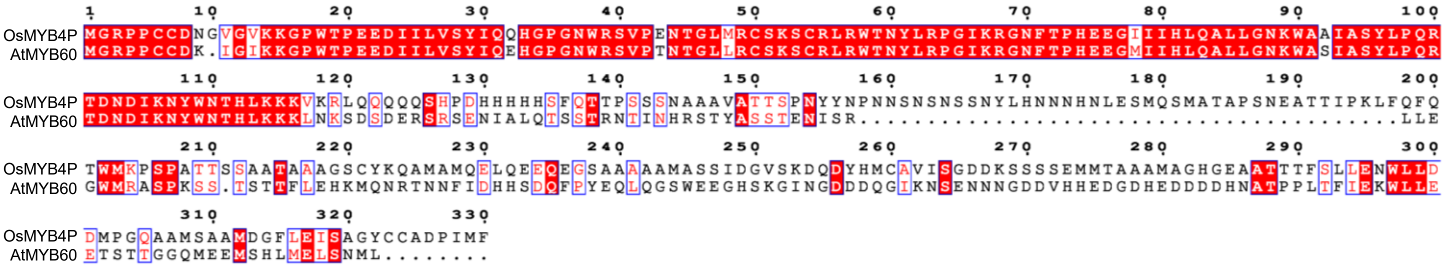
1 10 20 30 40 50 60 70 80 90 100
OsMYB4P MGRPPCCDNGVGVKKGPWTPEEDIILVSYIQQHGPGNWRSVPENTGLMRCSKSCRLRWTNYLRPGIKRGNFTPHEEGIIIHLQALLGNKWAAIASYLPQR
AtMYB60 MGRPPCCDK.IGIKKGPWTPEEDIILVSYIQEHGPGNWRSVPTNTGLLRCSKSCRLRWTNYLRPGIKRGNFTPHEEGMIIHLQALLGNKWASIASYLPQR
110 120 130 140 150 160 170 180 190 200
OsMYB4P TDNDIKNYWNTHLKKKVKRLQQQQQSHPDHHHHHSFQTTPSSSNAAAVATTSPNYYNPNNSNSNSSNYLHNNNHNLESMQSMATAPSNEATTIPKLFQFQ
AtMYB60 TDNDIKNYWNTHLKKKLNKSDSDERSRSENIALQTSSTRNTINHRSTYASSTENISR.......................................LLE
210 220 230 240 250 260 270 280 290 300
OsMYB4P TWMKPSPATTSSAATAAGSCYKQAMAMQELQEEQEGSAAAAAMASSIDGVSKDQDYHMCAVISGDDKSSSSEMMTAAAMAGHGEAATTTFSLLENWLLD
AtMYB60 GWMRASPKSS.TSTTFLEHKMQNRTNNFIDHHSDQFPYEQLQGSWEEGHSKGINGDDDQGIKNSENNNGDDVHHEDGDHEDDDDHNATPPLTFIEKWLLE
310 320 330
OsMYB4P DMPGQAAMSAAMDGFLEISAGYCCADPIMF
AtMYB60 ETSTTGGQMEEMSHLMELSNML........
